# Structural switching in self-assembled metal–ligand helicate complexes *via* ligand-centered reactions†

**DOI:** 10.1039/c6sc01038e

**Published:** 2016-03-29

**Authors:** Lauren R. Holloway, Hannah H. McGarraugh, Michael C. Young, Watit Sontising, Gregory J. O. Beran, Richard J. Hooley

**Affiliations:** a University of California – Riverside, Department of Chemistry and the UCR Center for Catalysis Riverside CA 92521 USA richard.hooley@ucr.edu

## Abstract

Ligand centered reactions are capable of conferring structural switching between a metastable, self-assembled Fe–iminopyridine aggregate and a stable M_2_L_3_ helicate. The reactivity is directed and accelerated by the stability of the final product structure. Under aerobic conditions, both substitution and oxidation occurs at the ligand, exploiting atmospheric oxygen as the oxidant. In the absence of air, reaction occurs more slowly, forming the less stable substitution product. Control ligands show a preference for simple substitution, but the self-assembly directs both substitution and oxidation. The metastable nature of the initial aggregate species is essential for the reaction: while the aggregate is “primed” for reaction, other analogous helicate structures are “locked” by self-assembly, preventing reactivity.

## Abstract 2

Ligand centered reactions are capable of conferring structural switching between a metastable, self-assembled Fe–iminopyridine aggregate and a stable M_2_L_3_ helicate.

## Graphical abstract

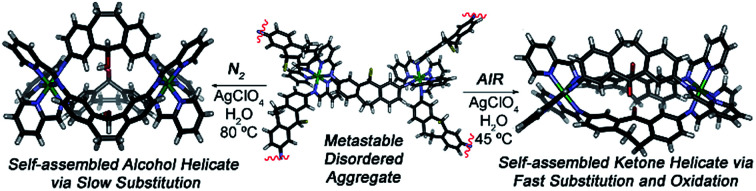

Allostery is a dominant mechanism of structural control in biosystems.^1^ A molecular recognition event, or structural change at one position in a biomacromolecule causes multiple small changes to build up in the larger structure. This causes a change in conformation elsewhere in the system, often activating or deactivating an enzyme by opening or closing an active site.^2^ Synthetic chemists have mimicked this effect to create allosteric catalysts^3^ and host molecules.^4^ The concept of controlling structure *via* remote reaction or recognition processes has also been applied in the construction of molecular machines and switches.^5^ Controlling these structural conversions requires the system to undergo a simple, predictable change in conformation upon a reaction in the body of the molecule. This field is dominated by rotaxanes and catenanes, as large changes in structure can be induced by simple mild reactions, often *via* redox^6^ or acid/base processes.^7^

Self-assembled metal–ligand cage complexes are an enticing target for this concept: by performing a reaction on the body of the cage, a change in ligand geometry could be induced that allows variation in host properties, guest release or other modes of analyte sensing. There are some elegant examples of this concept with metal-containing macrocycles,^8^ and numerous groups have exploited guest induced transformations,^9^ but simply conferring reactivity on a reversibly self-assembled cage complex is extremely challenging,^10^ let alone applying that reactivity to confer structural changes.^11^ Controlling the structure and assembly properties of complex, reversible self-assembled cages *via* external stimulus can extend the applications of these already valuable systems. Here we show that small changes in the non-coordinating backbone portion of a coordinating ligand can have drastic changes in the self-assembly properties of the cage complex, and the reaction outcome can be driven by the self-assembly process.

We have previously showed that rigid ligands based on dibenzosuberone^10*f*,12^ or fluorenone scaffolds^13^ can confer exquisite control on the structural and stereochemical outcomes of self-assembly. Small changes in backbone rigidity and variations in internal hydrogen-bonding have large effects on the self-assembly process, and can lead to self-sorting between similar ligand scaffolds,^12^ as well as stereoselective discrimination between many different isomers upon assembly.^13^

Most relevant to this discussion are the properties of substituted dibenzosuberone-based systems. Specifically, a distinct hierarchy in the stability of self-assembled M_2_L_3_ helicate structures is observed, dependent on the non-coordinating functional groups present on the ligand backbone (Fig. 1).^12^ The helicates are formed by multi-component self-assembly between 3,7-diaminosuberone-based species such as ketone A or alcohol B with 2-formylpyridine (PyCHO) and Fe(ClO_4_)_2_ in acetonitrile solvent. Complete selectivity for homocomplex formation was observed when these ligands were assembled in competition with each other, and suberone helicate 1 was formed with complete selectivity in the presence of ligand B.^12^ The significant stabilization of 1 over the highly similar 2 suggested that this product could provide directing effects in a postsynthetic modification process.

**Fig. 1:**
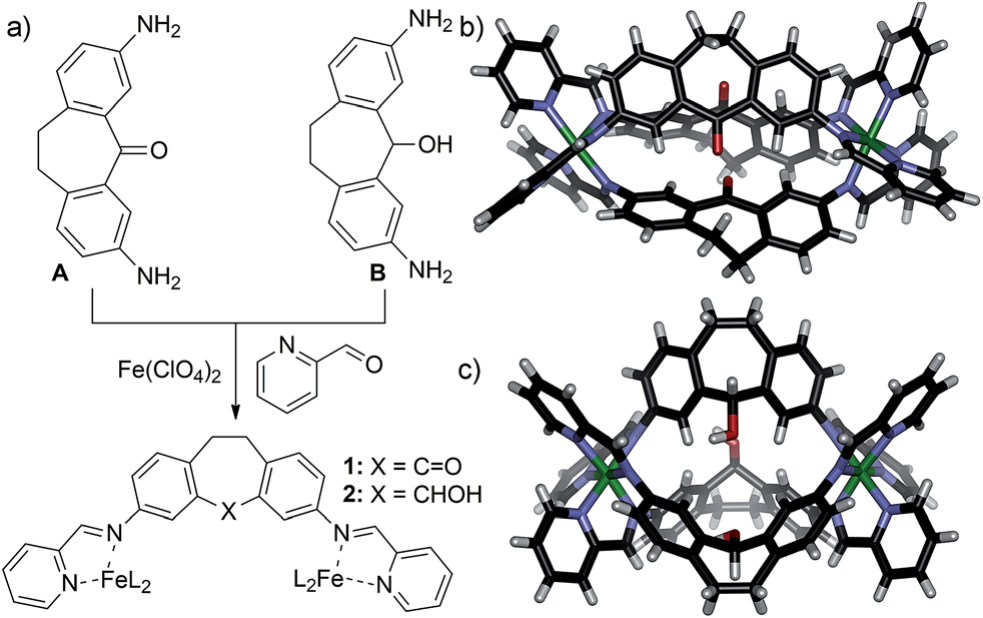
(a) Self-assembly of 3,7-diaminodibenzosuberone A and 3,7-diaminodibenzo-suberol B into helicates 1 and 2 respectively; structure of (b) 1 and (c) 2, as determined by X-ray diffraction analysis.^10f^

The core diaminosuberol scaffold B is easily accessed in 3 steps from dibenzosuberone,^10*f*^ and provides the foundation for the introduction of reactive functional groups to the self-assembled system that allow for postsynthetic modification. The challenge in postsynthetic modification of reversibly formed cage structures is tailoring the reaction conditions so that the sensitive self-assembled complexes are not destroyed or undergo unwanted side reactions during the process. The presence of nucleophiles is the greatest limitation: the cationic Fe–iminopyridine centers are sensitive to coordinative displacement, and are intolerant to species as mild as bromide or hydroxide ion, as well as primary amines or hydride reducing agents.

The choice of postsynthetic reaction is key, as is the nature of the reactive internal functionality. We have synthesized a variety of functionalized ligand cores containing internal groups but many of these groups are either too reactive to remain intact upon the initial self-assembly process, disrupt the assembly (*e.g.* amines) or are unreactive under mild conditions (*e.g.* ethers, carbamates^10*f*^). An alkyl chloride at the internal site is an enticing target for reaction, as suberyl cation formation can be forced by treatment with Ag^+^ cations, and the subsequent attack can be performed by weak, neutral nucleophiles that would not disrupt the Fe–iminopyridine assembly contacts.

Diamino suberyl chloride ligand C was synthesized in 64% yield from suberol B by selective protection of the amine groups, followed by chlorination and deprotection in conc. HCl. These conditions are mild enough that the reactive suberyl chloride group remains intact. Treatment of ligand C with 0.66 eq. Fe(ClO_4_)_2_ and 2 eq. PyCHO rapidly gave the characteristic purple color of an Fe–iminopyridine-based assembly product. However, NMR analysis of the product formed after 24 h heating was not representative of the expected M_2_L_3_ helicate (Fig. 2b). Instead of the usual sharp peaks observed in the ^1^H spectrum of Fe–iminopyridine assemblies,^9,10,13*a*,14^ broad mounds were observed throughout the aromatic and alkyl regions of the spectrum. Increased reaction time or temperature did not appreciably alter the obtained spectrum. No significant spectral differences were observed upon acquisition at elevated temperature, suggesting that the broad peaks in the NMR are not due to paramagnetism, but that the self-assembled product is not a single discrete complex. The spectrum is consistent with other disordered assemblies we have previously seen upon either incomplete assembly of mixed diaminosuberone species,^12^ or the untemplated assembly of diaminofluorenone derivatives.^13*a*^ Additionally, peaks corresponding to the diamino suberyl chloride or 2-formylpyridine starting materials were absent, suggesting that chloride ligand C is unable to form the expected M_2_L_3_ assembly, and instead forms an undefined oligomeric aggregate (Fig. 2a and b).

**Fig. 2:**
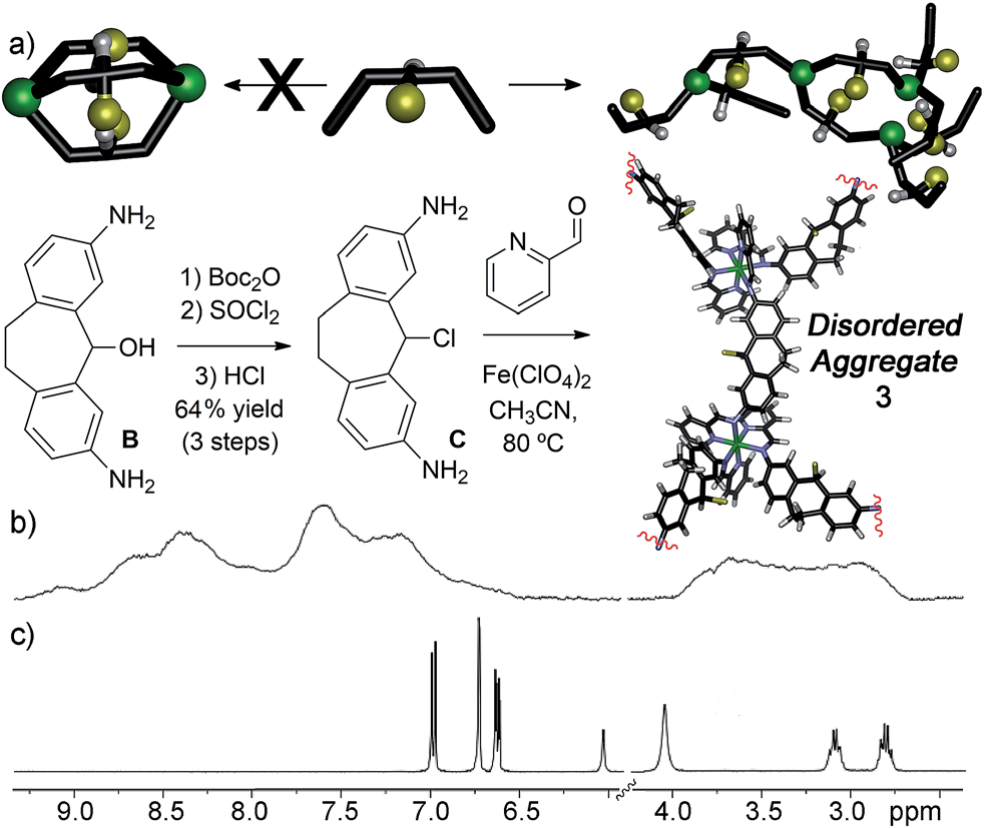
(a) Synthesis of diamino suberyl chloride ligand C and self-assembly into disordered aggregate 3. ^1^H NMR spectrum of (b) aggregate 3; (c) suberyl chloride ligand C (400 MHz, CD_3_CN, 298 K).

The lack of defined structure upon assembly of chloro ligand C is an advantage for testing reactivity: the metastable assembly provides a local minimum for postsynthetic modification,^14^ one that is more easily converted to a cage/helicate complex than a more thermodynamically stable and rigid system. For example, undefined fluorenol-based Fe–iminopyridine aggregates can be converted to M_4_L_6_ cages *via* anion templation.^13*a*^ To probe the reactivity of the chloro aggregate 3, a simple substitution reaction using water was attempted. One molar equivalent of both water and silver perchlorate was added to a solution of 3 in anhydrous CD_3_CN, and the sample heated in an NMR tube in air at 45 °C for 20 h. Silver perchlorate was used to drive a dissociative substitution process, enabling the use of a weak nucleophile. After 8 h, peaks corresponding to a discrete cage complex can be observed amongst the broad mounds corresponding to assembly 3. By 16 h, the NMR spectrum sharpened considerably, and a single product predominated (Fig. 3b and ESI†), with full completion observed at 20 h. Surprisingly, the observed peaks did not correspond to those for suberol helicate 2, the expected substitution product, but of ketone helicate 1, the product of both substitution and oxidation.

**Fig. 3:**
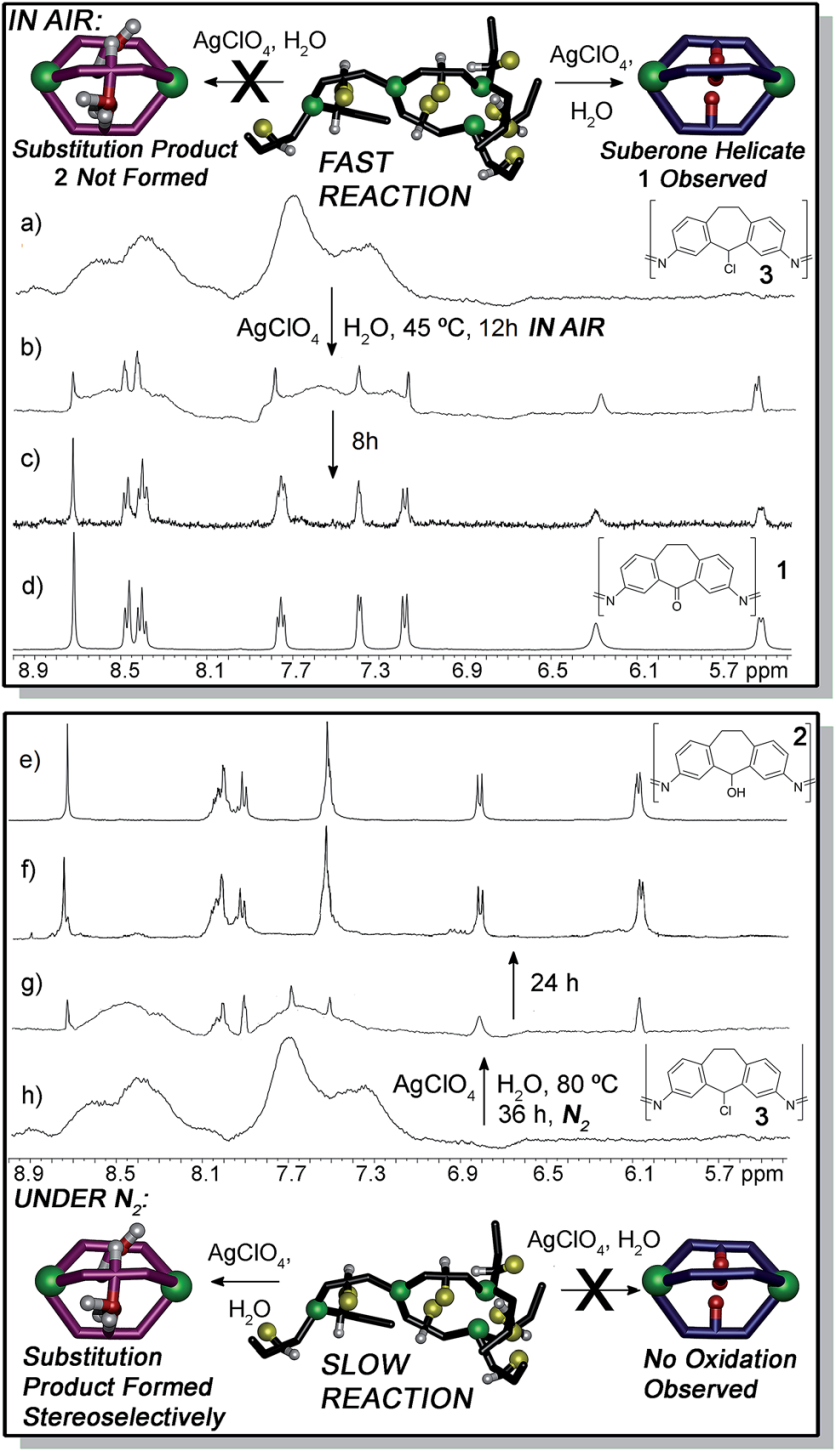
Structural switching upon ligand reaction. ^1^H NMR spectra (400 MHz, CD_3_CN, 298 K) of (a) aggregate 3; (b) 3 + AgClO_4_ + H_2_O, air, 45 °C, 12 h; (c) 3 + AgClO_4_ + H_2_O, air, 45 °C, 20 h; (d) suberone helicate 1; (e) suberol helicate 2; (f) 3 + AgClO_4_ + H_2_O, N_2_, 80 °C, 60 h; (g) 3 + AgClO_4_ + H_2_O, N_2_, 80 °C, 36 h; (h) aggregate 3.

As can be seen from Fig. 3b and c, as well as in the ESI,† only one type of cage is formed in the reaction, as the broad peaks for the chloro aggregate 3 smoothly give way to sharp peaks for suberone helicate 1. There is no buildup of other sharp peaks that would be indicative of other cage formation (see below for an example of NMR spectra of stereoisomeric mixtures of helicate assemblies), be they suberol helicate 2, suberone/suberol/suberyl chloride mixed heterocomplexes or other cage complexes of varying stoichiometries (*e.g.* M_4_L_6_ tetrahedra).

The reaction requires elevated temperature: reaction at 23 °C is extremely sluggish, and essentially no conversion is observed after 36 h. The specific silver salt and solvent were unimportant: use of AgNO_3_, or reaction in DMSO gave identical products under similar conditions. Most notably, at no point were any peaks observed that would correspond to the fully self-assembled suberol helicate 2. As only one discrete species is observed in the reaction, the nature of the reactive intermediates is unclear. It is clear, however, that there is no appreciable concentration of self-assembled helicate structures other than the final product (although small concentrations of intermediates must be present). The metastable chloro assembly 3 must give way to other assemblies, presumably containing suberol and/or suberone ligands, but only after oxidation does the final helicate 1 form. ESI-MS analysis of the mixture formed after 12 h reaction (see ESI†) showed small traces of a mixed chloroketone ML_3_ assembly, but the spectrum was dominated by cage 1 and its fragments.

The absence of the expected suberol helicate 2 in the presence of air is unusual: there are only mild oxidizing agents in the system (Ag ions, perchlorate ion and atmospheric oxygen), and the oxidation runs cleanly at only 45 °C. Also, the Fe^II^ centers are unaffected by the oxidation process. Ligand-centered redox processes on self-assembled cage complexes employing redox active metals as structural components are rare, and most cases of redox reactions on self-assembled cages naturally focus on metal-centered oxidations.^15^ To shed light on this, we attempted the reaction in the absence of air. If 3 was heated at 45 °C with AgClO_4_ and H_2_O under a N_2_ atmosphere, no reaction occurred after 48 h. At 80 °C, however, the reaction proceeded sluggishly to form suberol helicate 2. Only after 60 h reflux was complete conversion observed (Fig. 3f and g). Notably, the air-mediated oxidation process only occurs on the metastable assembly. If suberol helicate 2 was reacted with AgClO_4_ and H_2_O in air at 45 °C, no reaction occurred. Heating at 80 °C for 36 h also gave no conversion to suberone 1. Surprisingly, the suberol helicate was resistant to more stringent oxidative processes: heating 2 at 45 °C or 80 °C with Dess–Martin periodinane did not confer oxidation to suberone 1. The process does not appear to be acid-mediated, as addition of even stoichiometric amounts of acid to the reaction cause cage decomposition. Slight decomposition was observed, but the helicate remained mostly intact, and no Fe-based oxidation occurred. Evidently, reaction of 3 is facile, but once a favorable self-assembled cage is formed, the reactive carbon center of the system becomes “locked”, and resistant to further reaction. The metastable assembly 3 is “primed” for reaction, whereas the helicates are not.

Both the metastable reactant assembly and thermodynamically favored helicate product are essential for optimal reaction. As an illustration of this, control experiments were performed with stable ligand surrogate, 3,7-diacetamidosuberylchloride 6. Control 6 was chosen to mimic the electronic nature of the constituent amino-pyridine ligand of 3, while preventing amine or imine-based solvolyses or other unwanted side reactions. Chloride 6 was highly resistant to dissolution in acetonitrile, so the control reactions were performed in DMSO-*d*_6_, a solvent that also allowed smooth conversion of 3 to suberone 1. When 6 was exposed to the same conditions that gave complete oxidation of 3 to helicate 1 (*i.e.* 1 eq. AgClO_4_ and 1 eq. H_2_O, heating at 45 °C for 24 h), no reaction was observed. Only when the temperature was increased to 80 °C and the reaction time increased to 36 h did any new products form, and in this case, only the alcohol substitution product 7 formed: no evidence of any oxidation to ketone was seen, even under these harsher conditions (Fig. 4). The substitution reaction proceeds normally with or without air. These conditions are similar to those needed for the air-free substitution reaction of 3, indicating that the accelerated oxidation reaction of 3 in air is funneled to the most stable suberone product, which provides a directing effect on the reaction outcome. Other tests were performed with control ligand 6 (see ESI†). In the absence of silver (employing Bu_4_NClO_4_ instead) or the absence of ClO_4_^−^ anion (using AgNO_3_), no reaction was observed, even after extensive heating. Tests performed under strictly anhydrous conditions also (unsurprisingly) gave no reaction. Notably, even in the presence of added Fe^II^ salts, the main product was the alcohol rather than the ketone.

**Fig. 4:**
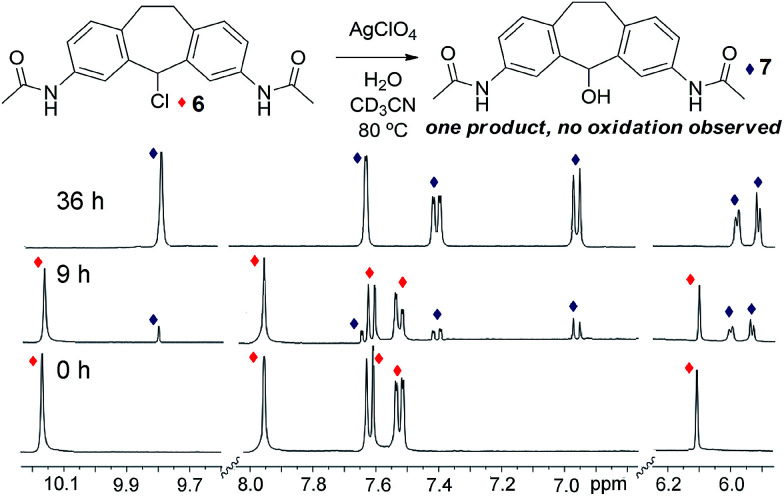
^1^H NMR spectra of control ligand 6, and after reaction with 1 eq. H_2_O and 1 eq. AgClO_4_ at 80 °C for 9 h and 36 h with conversion to ligand 7 (400 MHz, CD_3_CN, 298 K).

The metastable aggregate 3 is essential for reactivity, but is also somewhat unique: we have made numerous other diamino suberyl scaffolds that each form M_2_L_3_ helices upon multicomponent self-assembly. To investigate the reasons why ligand C is incapable of forming a stable self-assembled cage, and to shed light on the driving forces for the reactive behavior of 3, we synthesized two more analogs of ligand C that mimic the size and reactive nature of the chloro group in C. Mesylate ligand D was synthesized from alcohol ligand B*via* the same protection/activation/deprotection route as C. This provides a leaving group to the central scaffold, but one that cannot be forced towards cation formation *via* the application of Ag^+^ ions. Trifluoroethylether ligand E was synthesized directly from B*via* treatment with acid in trifluoroethanol, and provides a less reactive, yet sterically similar internal group.

Our initial explanation for the uncontrolled assembly of C was the lack of hydrogen bonding groups in the central core. Suberol cage 2 exhibits a preference for a single (all-in) isomer at the prochiral CHOH stereocenters, likely due to self-complementary hydrogen bonding upon assembly.^10*f*^ Both ligands D and E have no H-bonding groups, but smoothly form M_2_L_3_ helicate structures. Unlike the alcohol cage 2, no stereocontrol was observed in the assembly. The spectral data for trifluoroether cage 5 is shown in Fig. 5, and illustrate the type of spectra observed for isomerically impure M_2_L_3_ helicates. More peaks were present than would be expected for a single isomer, however the peaks are all sharp and well-defined, unlike the spectra seen for 3. DOSY analysis shows that the multiple peaks present in the ^1^H NMR all belong to one species of the same size. ^19^F NMR for the trifluoroether cage shows several triplets indicating that the trifluoroether group is subjected to several different environments from the various isomeric combinations of orienting the group in and out of the cage. Mesylate cage 4 also showed no stereocontrol upon assembly, and gave similar mixtures of isomers in its NMR spectra. The similarity in structure between C, D and E is incongruous with their large differences in assembly properties. Attempts to synthesize the analogous bromo- and iodo-suberyl core analogs were unsuccessful due to the reactivity of the doubly benzylic halides, so we turned to computational analysis. The structure of each possible isomer of the M_2_L_3_ helicate complexes that could be formed upon self-assembly of ligand C, Fe(ClO_4_)_2_ and PyCHO was optimized using dispersion-corrected density functional theory (at the ωB97X-D/6-31G* level, see ESI† for details), and the energies were compared to that of suberone complex 1. The calculated energy of each isomer of the chloride cage was significantly higher than that of 1, ranging from approximately +10 kcal mol^−1^ for the “all-out” isomer, to +30 kcal mol^−1^ for the “all-in” isomer. The M_2_L_3_ helicates have very small cavities, and are highly sensitive to interactions between large atoms and the other ligand backbones in the assembly. The structure optimizations indicated that the Cl atom is too large to allow assembly due to steric clashes with the other ligand backbones. Evidently, smaller atoms such as O are tolerable, and the extra substituents in 4 and 5 can point away from the core, allowing smooth M_2_L_3_ formation.

**Fig. 5:**
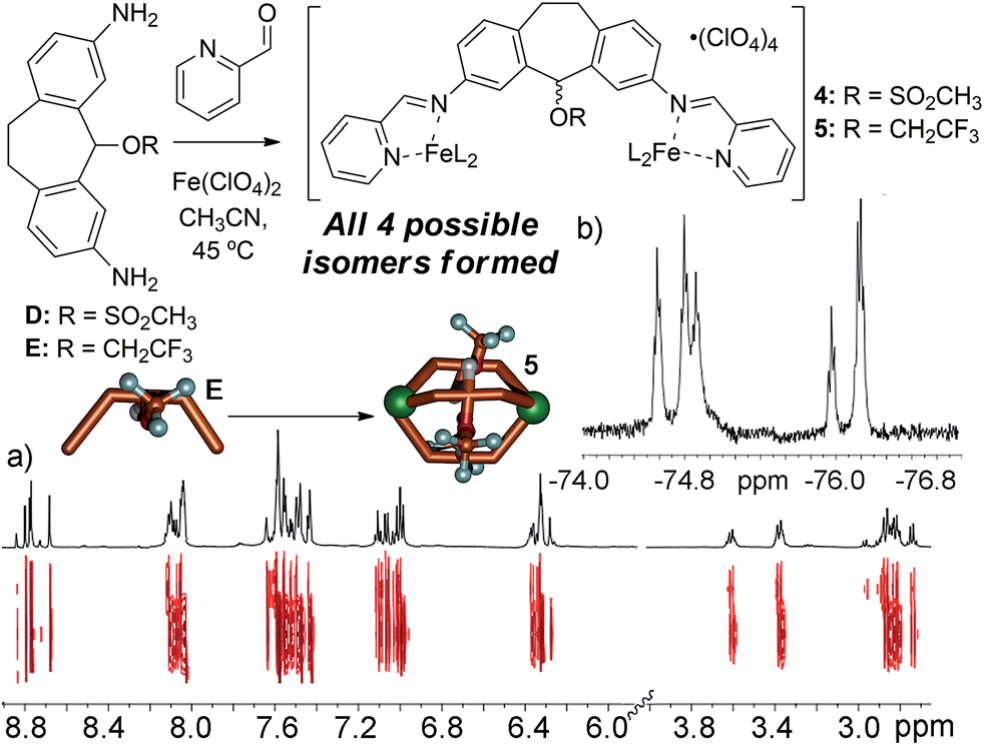
(a) ^1^H and 2D DOSY NMR spectra of cage 5; (b) ^19^F NMR spectrum of cage 5 (600 MHz, CD_3_CN, 298 K), showing multiple isomers.

Chloride abstraction and cation formation on the ligand is essential for the reactivity of 3, and neither mesylate cage 4 nor trifluoroether cage 5 showed any reactivity. When either 4 or 5 was heated at 45 °C or 80 °C with H_2_O and AgClO_4_, in either CD_3_CN or DMSO-d_6_, no change in the NMR spectra was observed after 36 h. Neither the mesylate nor the trifluoroethyl ether group can be abstracted by Ag^+^ ions, and so an associative reaction would be required. The hindered nature of the central carbon atom in helicates 4 and 5 evidently limits access to reactants, and only upon cation formation can reaction occur. Easier access to the central carbon atom, and the metastable nature of 3 are responsible for its unique behavior.

In conclusion, we have shown that ligand centered reactivity can confer structural switching between a metastable self-assembled aggregate species and a stable M_2_L_3_ helicate structure. The outcome of this process is directed and accelerated by the stability of the final product structure: suberone helicate 1 is formed preferentially under aerobic conditions, and the process employs atmospheric oxygen as the oxidant for ligand-centered reaction in the presence of multiple redox-active coordinating metal ions. In the absence of air, the switching process occurs more slowly, and the reaction is directed to the less stable product of simple substitution, suberol helicate 2. In this case, the reaction is stereocontrolled, and one isomer of product is formed, directed by internal hydrogen bonding. In the absence of any directing effects from self-assembly, no oxidation is observed, even under harsh conditions and the presence of air: the control ligand shows a preference for simple substitution, but the self-assembly directs both substitution and oxidation. The metastable nature of the initial aggregate species is essential for the reaction: while aggregate 3 is “primed” for reaction, other analogous, putatively reactive helicate structures are inert to both substitution and oxidation, as the self-assembly “locks” the system, preventing reactivity.

## Supplementary Material

SC-007-C6SC01038E-s001
