# Supplementary material for: Structural switching in self-assembled metal–ligand helicate complexes via ligand-centered reactions
Source: Chem Sci. 2016 Mar 29;7(7):4423–7. doi: 10.1039/c6sc01038e (PMC6014161; doi:10.1039/c6sc01038e)
Supplement: SC-007-C6SC01038E-s001 [file SC-007-C6SC01038E-s001.pdf]

Electronic Supplementary Material (ESI) for Chemical Science.
This journal is © The Royal Society of Chemistry 2016

Structural Switching in Self-Assembled Metal-Ligand Helicate Complexes via Ligand-Centered Reactions

Lauren R. Holloway, Hannah H. McGarraugh, Michael C. Young, Watit Sontising, Gregory J. O. Beran and Richard J. Hooley*

Department of Chemistry, University of California, Riverside, CA 92521.

richard.hooley@ucr.edu

Electronic Supplementary Information

Table of Contents

General Information.. 2
Synthesis of Compounds 2
NMR Spectral Data 9
Mass Spectral Data 23
Cage Oxidation Experiments 24
Control Experiments 34
Structure Optimizations.. 41
References 43

General Information

^{1}H and ^{13}C spectra were recorded on a Varian Inova 400 MHz or Varian Inova 500 MHz NMR spectrometer. DOSY spectra were recorded on a Bruker Avance 600 MHz spectrometer. Proton (^{1}H) chemical shifts are reported in parts per million (δ) with respect to tetramethylsilane (TMS, δ=0), and referenced internally with respect to the protio solvent impurity. Deuterated NMR solvents were obtained from Cambridge Isotope Laboratories, Inc., Andover, MA, and used without further purification. Mass spectra were recorded on an Agilent 6210 LC TOF mass spectrometer using electrospray ionization with fragmentation voltage set at 115 v and processed with an Agilent MassHunter Operating System. All other materials were obtained from Aldrich Chemical Company, St. Louis, MO, or TCI, Tokyo, Japan and were used as received. Solvents were dried through a commercial solvent purification system (Pure Process Technologies, Inc.). Molecular modeling (semi-empirical calculations) was performed using the AM1 force field using SPARTAN.[1] Helicates **1** and **2** were prepared according to our previous report,[2] which contains full characterization.

Synthesis of Compounds

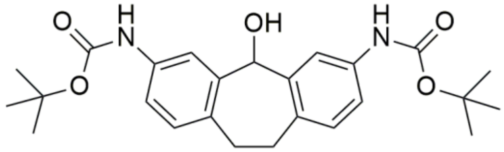

Dibenzosuberol-3,7-di-tert-butylcarbamate (*S-1*):

3,7-Diaminodibenzosuberol[2] (350 mg, 1.46 mmol) was added to a 50 mL round bottom flask with a stir bar, followed by the addition of DMA (10 mL) and trimethylamine (0.61 mL). Di-tert-butyl dicarbonate (954 mg, 4.37 mmol) was slowly added to the flask. The reaction mixture was stirred at room temperature for 36 h, after which the reaction mixture was poured into a separatory funnel and washed with 3 x 10 mL portions of hexane. The reaction layer was then slowly dropped into a flask containing 100 mL of ice water with rapid stirring. The precipitate that formed was filtered, dried and collected as a tan solid (604 mg, 95 %). ^{1}H NMR (400 MHz; DMSO-d_6) δ 9.36 (s, 2H), 7.69 (s, 2H), 7.23 (d, 2.1 Hz, 2H), 6.88 (d, J = 2.1 Hz, 2H), 5.92 (d, J = 1.9 Hz, 1H), 5.83 (d, J = 1.9 Hz, 1H), 3.31 (m, 2H), 2.91 (m, 2H) 1.45 (s, 18H). ^{13}C NMR (100 MHz, DMSO-d_6): δ 168.6, 144.2, 137.72, 131.9, 130.18, 117.9, 115.8, 79.8, 69.9, 31.5, 24.6. HRMS (ESI) m/z calcd. for $C_{25}H_{31}N_2O_5$ ($[M-H]^-$) 439.2238, found 439.1937.

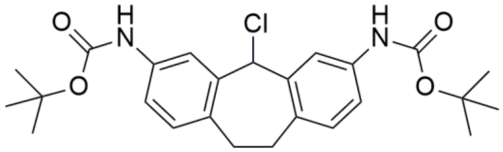

Dibenzosuberylchloride-3,7-ditertbutylcarbamate (*S-2*)

S-1 (100 mg, 0.23 mmol) was placed in a 25 mL round bottomed flask with a stir bar followed by 5 mL of dry DCM. One equivalent of sodium hydride (9 mg, 0.22 mmol) was slowly added to the flask and allowed to stir for 4 h. Thionyl chloride (0.016 mL, 0.22 mmol) was slowly added to the flask and the reaction was allowed to stir for 24 h at 45 °C. The reaction mixture was then filtered and the solvent removed *in vacuo* to yield a pale orange solid (77 mg, 74%). The product was purified by column chromatography using 15:85 ethyl acetate:hexane as the eluent. ^{1}H NMR (400 MHz; DMSO-d_6) δ 9.3 (s, 2H), 7.94 (d, 2.2Hz, 2H), 7.22 (dd, 8.3, 2.2 Hz, 2H), 7.18 (d, J = 8.3 Hz, 2H), 6.22 (s, 1H), 3.49 (m, 2H), 2.77 (m, 2H), 1.44 (s, 18H). ^{13}C NMR (100 MHz, DMSO-d_6): δ 167.8, 144.8, 140.12, 137.74, 130.9, 118.13, 115.61, 78.3, 60.7, 29.8, 24.2. HRMS (ESI) m/z calcd. for $C_{25}H_{31}N_2O_4Cl$ ([M]$^+$) 458.1993, found 458.1972.

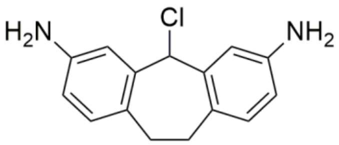

3,7- Diaminodibenzosuberylchloride (Dianiline C)

S-2 (100 mg, 0.22 mmol) was placed in a 25 mL round bottom flask with a stir bar followed by 4 mL of 2M HCl in THF. The reaction was stirred for 12 h at room temperature, then poured over 5g ice. The pH was brought to 7.5 using saturated sodium bicarbonate and the solid quickly filtered to yield product (49 mg, 86%). ^{1}H NMR (400 MHz, DMSO-d_6): δ 6.89 (d, *J* = 8.0 Hz, 2H), 6.63 (d, *J* = 2.5 Hz, 2H), 6.53 (dd, *J* = 8.0, 2.5 Hz, 2H), 5.96 (dd, 1H), 4,53 (s, 4H), 3.03 (m, 2H), 2.77 (m, 2H). ^{13}CNMR (100 MHz, DMSO-d_6): δ 142.6, 137.8, 131.7, 126.4, 124.3, 114.2, 67.1, 31.3. HRMS (ESI) m/z calcd. for $C_{15}H_{15}N_2Cl$ ([M+H]$^+$) 259.0124, found 259.0097.

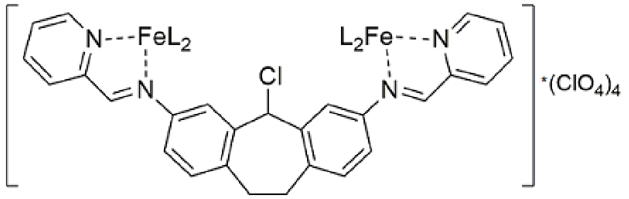

Metastable Aggregate 3

Dianiline C (100 mg, 0.39 mmol), 2-pyridine carboxaldehyde (74 µL, 0.78 mmol) and $Fe(ClO_4)_2{\bullet}H_2O$ (71.6 mg, 0.26 mmol) were combined in anhydrous MeCN (10 mL) in a 50 mL round-bottomed flask under a blanket of N_2, followed by heating to 45 °C for 24 h. The solution was then cooled to room temperature, diluted with Et_2O (30 mL), and cooled to -25 °C followed by filtration of the resulting precipitate. Drying product *in vacuo* gave product as a purple solid (169 mg, 98%). ^{1}H NMR and ^{13}C NMR: Undefined coordination aggregate. See Figures **S7**, **S8** and **S31**.

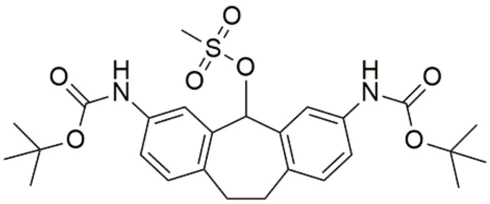

Dibenzosuberylmesylate-3,7-ditertbutylcarbamate (*S-3*)

S-1 (100 mg, 0.22 mmol) was placed in a 25 mL round bottomed flask with a stir bar followed by 5 mL of dry dichloromethane. One equivalent of sodium hydride 60% in mineral oil (9 mg, 0.22 mmol) was slowly added to the flask and allowed to stir for 6 h. Mesyl chloride (0.018 mL, 0.22 mmol) was slowly added to the mixture and the reaction was allowed to stir at room temperature for an additional 6 h. The reaction mixture was placed in a separatory funnel and washed with water (2 x 5 mL) and the organic layer dried over $MgSO_4$. The solvent was removed *in vacuo* to yield product as a pale yellow solid (110 mg, 96 %). ^{1}H NMR (400 MHz; DMSO-d_6) δ 9.12 (s, 2H), 7.61 (s, 2H), 7.09 (d, 2.1 Hz, 2H), 6.9 (d, J = 2.1 Hz, 2H), 6.02 (s, 1H), 3.16 (m, 2H), 2.88 (m, 2H) 2.28 (s, 3H) 1.42 (s, 18H). ^{13}C NMR (100 MHz, DMSO-d_6): δ 168.6, 143.9, 135.32, 132.2, 129.8, 117.9, 115.8, 80.1, 70.1, 52.3, 32.5, 25.1. HRMS (ESI) m/z calcd. for $C_{26}H_{34}N_2O_7S$ ($[M]^+$) 518.2087, found 518.1994.

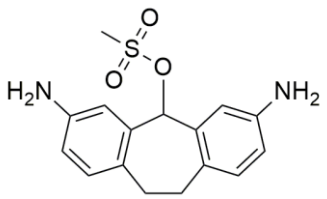

3,7- Diaminodibenzosuberylmesylate (Dianiline D)

S-3 (100 mg, 0.19 mmol) was placed in a 25 mL round bottom flask with a stir bar followed by 2 mL of trifluoroacetic acid. The reaction was stirred for 12 h at room temperature, then poured over 5g ice. The pH was brought to 7.5 using saturated sodium bicarbonate and the solid filtered to yield product (52 mg, 85 %). ^{1}H NMR (400 MHz, DMSO-d_6): δ 7.05 (d, J = 8.0 Hz, 2H), 6.64 (d, J = 2.5 Hz, 2H), 6.48 (dd, J = 8.0, 2.5 Hz, 2H), 6.24 (dd, 1H), 4.59 (s, 4H), 3.24 (s, 3H) 3.01 (m, 2H), 2.71 (m, 2H). ^{13}CNMR (100 MHz, DMSO-d_6): δ 142.6, 137.5, 127.1, 126.8, 116.3, 114.9, 78.1, 43.1, 34.9. HRMS (ESI) m/z calcd. for $C_{16}H_{19}N_2O_3S$ ($[M+H]^+$) 319.1012, found 319.0914.

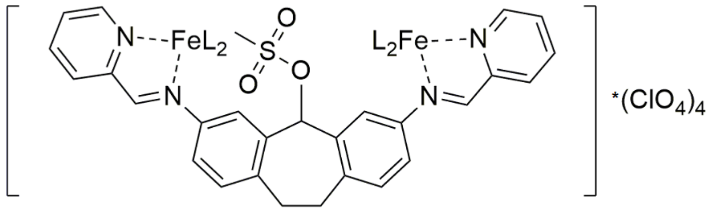

Mesylate Cage 4

Dianiline D (50 mg, 0.19 mmol), 2-pyridine carboxaldehyde (37 µL, 0.39 mmol) and $Fe(ClO_4)_2$•XH_2O (42 mg, 0.13 mmol) were combined in anhydrous MeCN (10 mL) in a 50 mL round-bottomed flask under a blanket of N_2, followed by heating to 45 °C for 24 h. The solution was then cooled to room temperature, diluted with Et_2O (30 mL), and cooled to -25 °C followed by filtration of the resulting precipitate. Drying product *in vacuo* gave product as a purple solid (84.5 mg, 98%). ^{1}H NMR and ^{13}C NMR: Complex mixture of stereoisomers.[2] See SI Figures **S13–S15.** HRMS (ESI) m/z calcd. for $C_{84}H_{72}Fe_2N_{12}O_{21}S_3Cl_3$ ($[Fe_2L_3$•$(ClO_4)_3]^+$) 1899.9137, found 1900.2734.

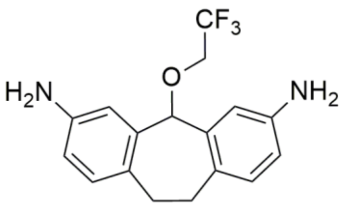

3,7-Diaminodibenzosuberyl-2,2,2-trifluoroethyl ether (Dianiline E):

3,7-diaminodibenzosuberol[2] (70 mg, 0.29 mmol) was added to 2,2,2-trifluoroethanol (3 mL), followed by addition of trifluoromethanesulfonic acid (250 μL, 2.8 mmol). The reaction mixture was stirred at room temperature for 70 h, followed by neutralization of the solution using saturated sodium bicarbonate. The resulting solid was filtered to give product as a white solid (79 mg, 84 %). ^{1}H NMR (400 MHz, DMSO-d_6): δ 6.76 (d, J = 8.0 Hz, 2H), 6.51 (d, J = 1.7 Hz, 2H), 6.40 (dd, J = 8.0, 1.7 Hz, 2H), 5.15 (s, 1H), 3.87 (q, J = 9.3 Hz, 2H), 3.11 (d, J = 6.7 Hz, 2H), 2.65 (d, J = 6.7 Hz, 2H). ^{13}CNMR (100 MHz, DMSO-d_6): δ 147.1, 138.3, 133.3, 131.5, 126.6, 123.8, 115.1, 80.1, 65.4, 31.6. HRMS (ESI) m/z calcd. for $C_{17}H_{18}N_2F_3O$ ($[M+H]^+$) 323.1366, found 323.1362.

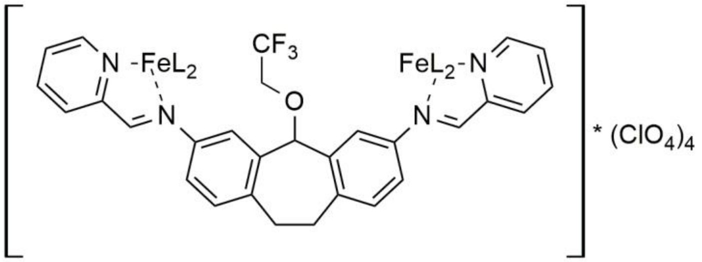

Trifluoroether Cage 5:

Dianiline **3** (100 mg, 0.31 mmol), 2-pyridine carboxaldehyde (59 μL, 0.62 mmol) and $Fe(ClO_4)_2 \cdot xH_2O$ (66.8 mg, 0.20 mmol) were combined in anhydrous MeCN (10 mL) in a 50 mL round-bottomed flask under a blanket of N_2, followed by heating to 45 °C for 24 h. The solution was then cooled to room temperature, diluted with Et_2O (30 mL), and cooled to -25 °C followed by filtration of the resulting precipitate. Drying product *in vacuo* gave product as a purple solid (166 mg, 96%). ^{1}H NMR and ^{13}C NMR: Complex mixture of stereoisomers.[2] See SI Figures **S19–S22**. HRMS (ESI) m/z calcd. for $C_{58}H_{46}Fe_2N_8O_{14}F_6Cl_3$ ($[Fe_2L_2 \cdot (ClO_4)_3]^+$) 1409.1018, found 1409.0489.

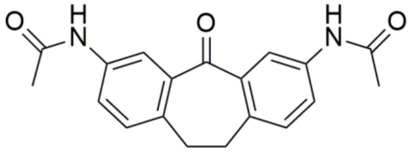

3,7-Diacetamidedibenzosuberone (*S-4*):

3,7-Diaminodibenzosuberone (200 mg, 0.84 mmol) was added to a 50 mL round bottom flask with stir bar, followed by the addition of THF (10 mL). Triethylamine (0.30 mL, 2.09 mmol) and 2.1 equivalents acetic anhydride (0.18 mL, 1.76 mmol) were added to the flask. The mixture was stirred at room temperature for 14 h, followed by pouring the resulting solution over 10 g ice. The resulting precipitate was filtered to give product as a pale yellow solid (270 mg, 89%). ^{1}H NMR (400 MHz, DMSO-d_6): δ 10.04 (s, 2H), 8.01 (d, *J* = 2.0 Hz, 2H), 7.73 (dd, *J* = 8.2, 2.1 Hz, 2H), 7.22 (d, *J* = 8.3 Hz, 2H), 3.03 (s, 4H), 2.01 (s, 6H). ^{13}CNMR (100 MHz, DMSO-d_6): δ 194.6, 169.0, 138.6, 138.4, 137.4, 130.7, 123.9, 120.8, 34.4, 24.6. HRMS (ESI) m/z calcd. for $C_{19}H_{18}N_2O_3$ ($[M+H]^+$) 323.1317, found 323.1362.

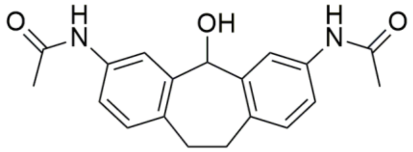

3,7-Diacetamidedibenzosuberol (*S-5*):

S-4 (200 mg, 0.62 mmol) was added to a 50 mL round bottom flask with stir bar, followed by addition of MeOH (10 mL). Sodium borohydride (23 mg, 0.62 mmol) was slowly added. The reaction was stirred at room temperature overnight. After 14 h the reaction mixture was poured over 15g of ice, neutralized with 1M HCl and allowed to stir for 1h. The solid was filtered, dried and collected as a tan solid (185 mg, 92 %). ^{1}H NMR (400 MHz; CD_3CN) δ 9.78 (s, 2H), 7.62 (d, *J* = 1.8 Hz, 2H), 7.39 (dd, *J* = 8.1, 2.0 Hz, 2H), 6.94 (d, *J* = 8.2 Hz, 2H), 6.0 (d, *J* = 4.0 Hz, 1H), 5.93 (d, *J* = 4.2 Hz, 1H), 3.15 (m, 2H), 2.90 (m, 2H), 1.96 (s, 6H). ^{13}C NMR (100 MHz, DMSO-d_6): δ 168.58, 144.20, 137.72, 131.90, 130.18, 117.97, 115.76, 69.96, 31.54, 24.58. HRMS (ESI) m/z calcd. for $C_{19}H_{19}N_2O_3$ ($[M-H]^-$) 323.1474, found 323.1501

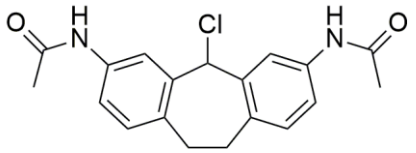

3,7-Diacetamidedibenzosuberylchloride (*S-6*)

S-5 (200 mg, 0.61 mmol) was placed in a 25 mL round bottomed flask with a stir bar followed by 5 mL of dry THF. One equivalent of sodium hydride (24 mg, 0.61 mmol) was slowly added to the flask and allowed to stir for 2 h. Thionyl chloride (0.045 mL, 0.22 mmol) was slowly added to the flask and the reaction was allowed to stir for 24 h. The reaction mixture was then poured over 20 mL of ice water and the tan solid filtered (163 mg, 78 %). ^{1}H NMR (400 MHz; CD_3CN) δ 10.16 (s, 2H), 7.94 (s, 2H), 7.61 (d, J = 8.3 Hz, 2H), 7.54 (d, J = 8.3 Hz, 2H), 6.11 (s, 1H), 3.17 (m, 2H) 2.9 (m, 2H), 2.02 (s, 6H). ^{13}C NMR (100 MHz, DMSO-d_6): δ 166.58, 136.20, 134.72, 131.50, 130.08, 117.77, 114.99, 57.86, 29.54, 24.38. HRMS (ESI) m/z calcd. for $C_{19}H_{19}N_2O_2Cl$ ($[M]^+$) 342.1135, found 342.1096.

NMR Spectral Data

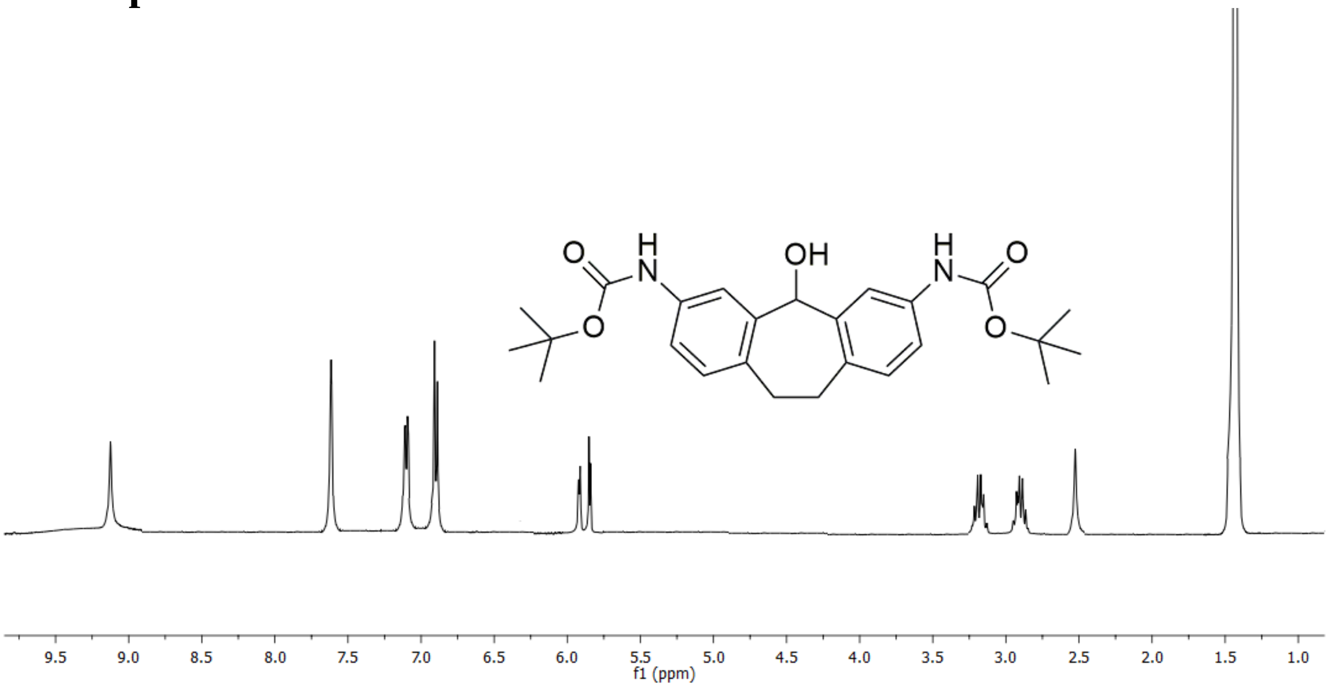

Figure S1. ^{1}H NMR spectrum of ***S-1*** (DMSO-d_6, 400 MHz, 298 K).

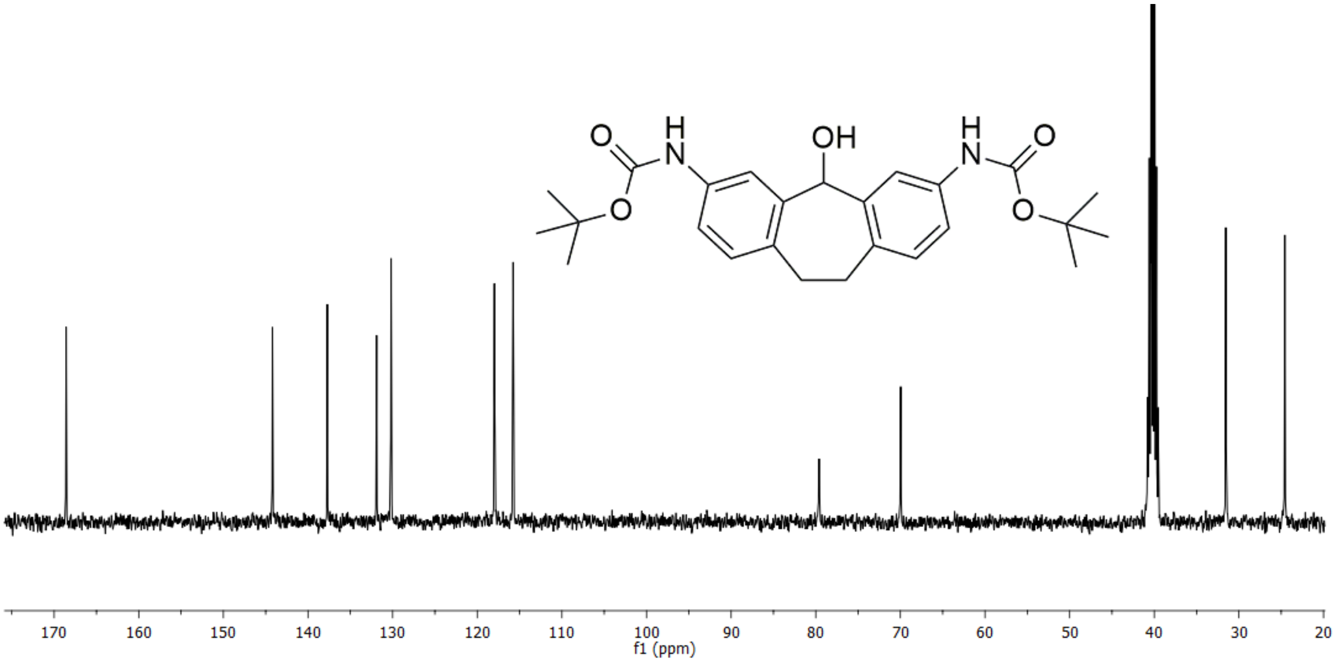

Figure S2. ^{13}C NMR spectrum of ***S-1*** (DMSO-d_6, 100 MHz, 298 K).

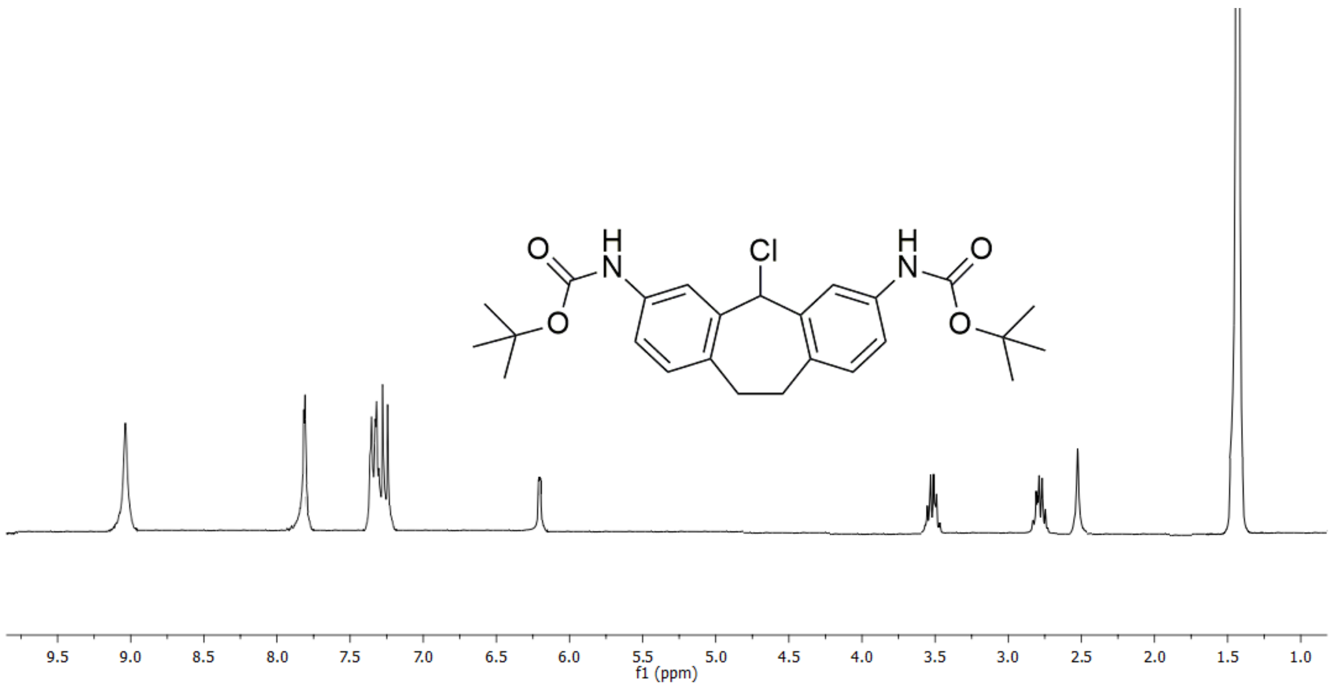

Figure S3. ^{1}H NMR spectrum of ***S*-2** (DMSO-d_6, 400 MHz, 298 K).

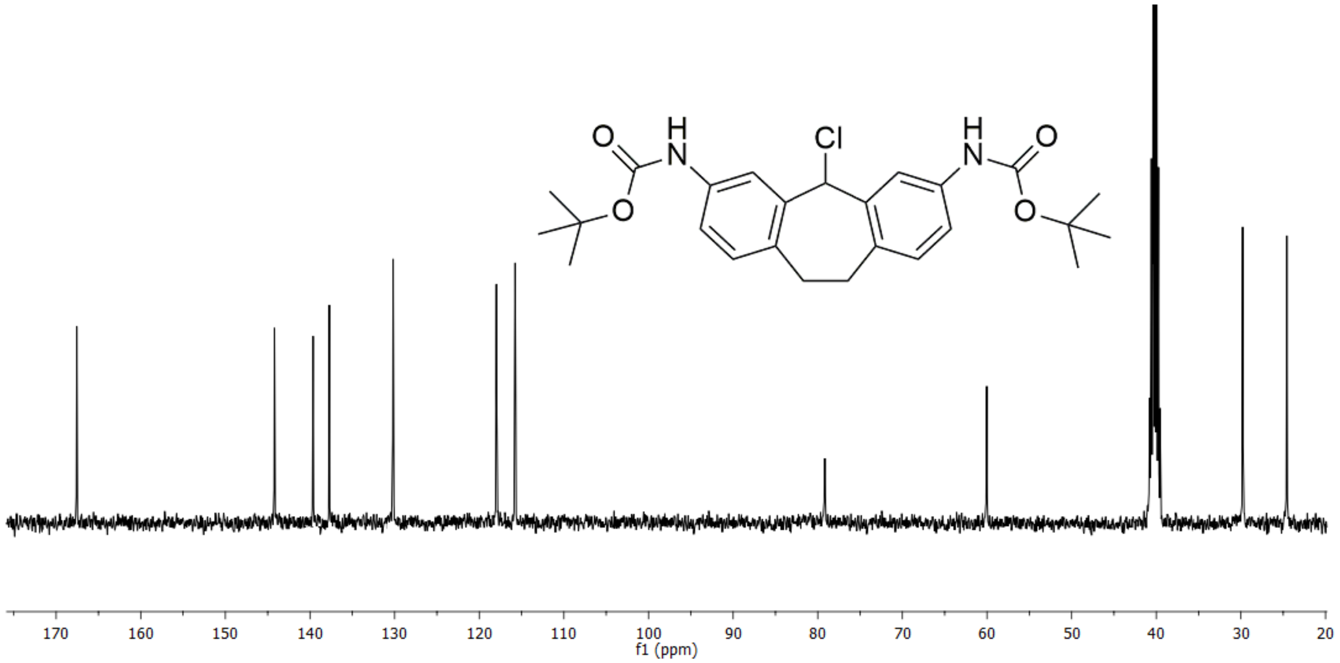

Figure S4. ^{13}C NMR spectrum of ***S*-2** (DMSO-d_6, 100 MHz, 298 K).

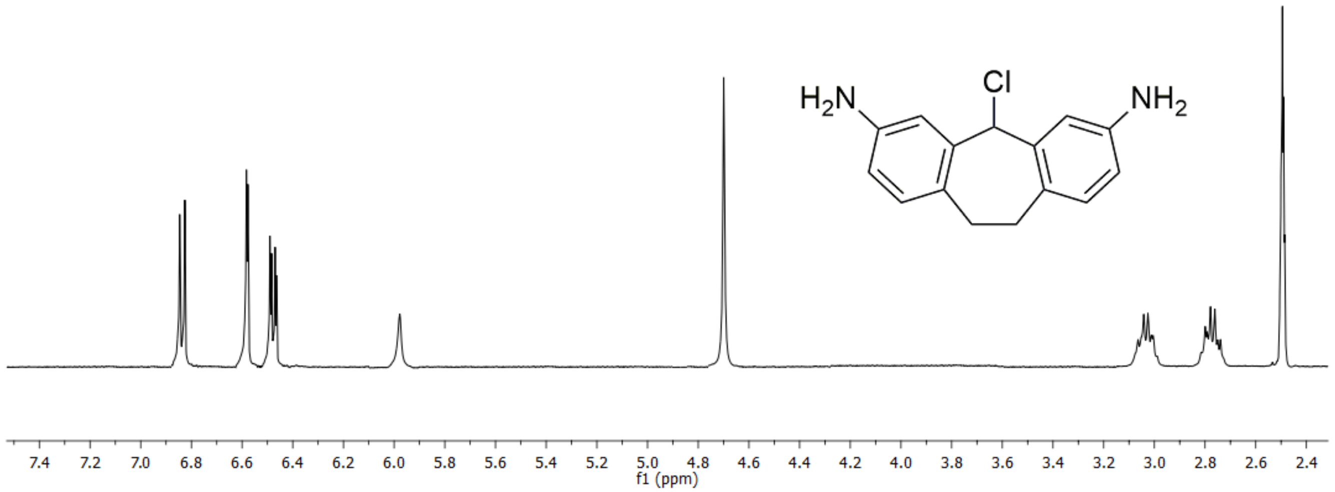

Figure S5. ^{1}H NMR spectrum of Dianiline **C** (DMSO-d_6, 400 MHz, 298 K).

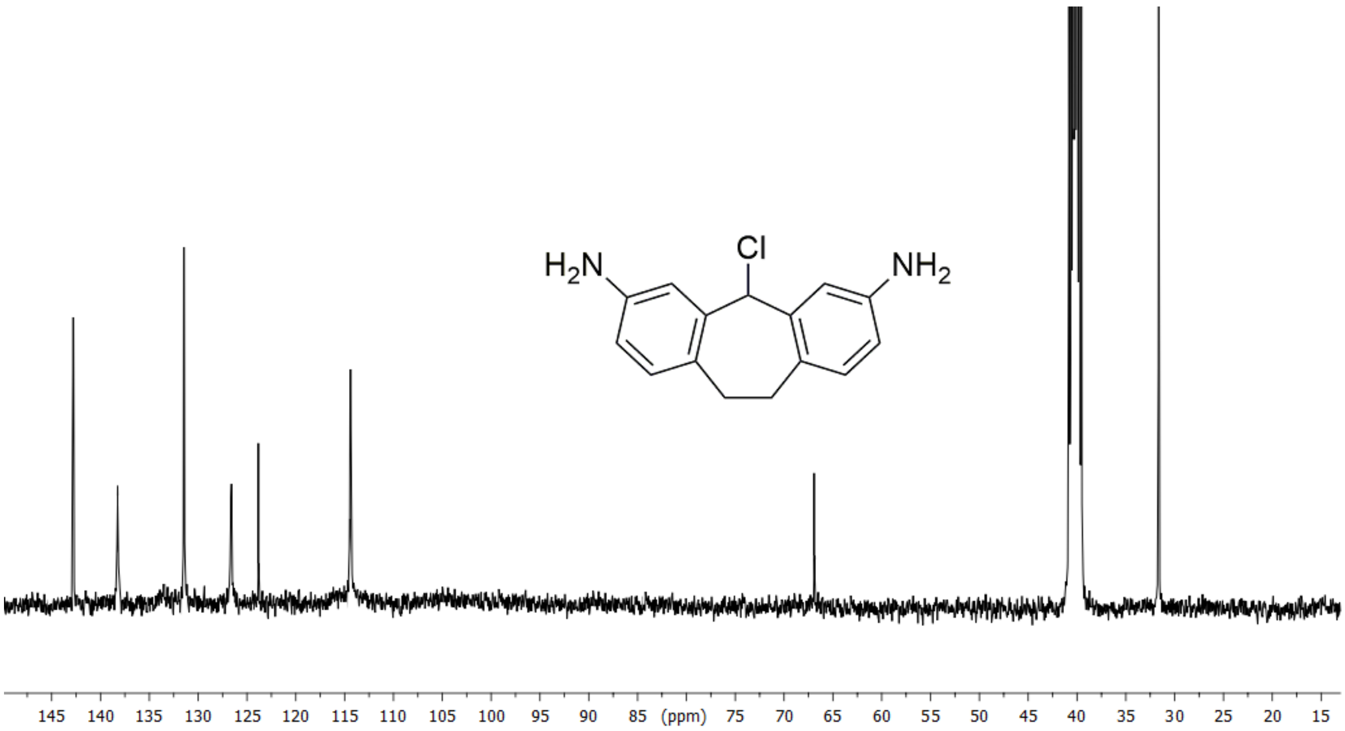

Figure S6. ^{13}C NMR spectrum of Dianiline **C** (DMSO-d_6, 100 MHz, 298 K).

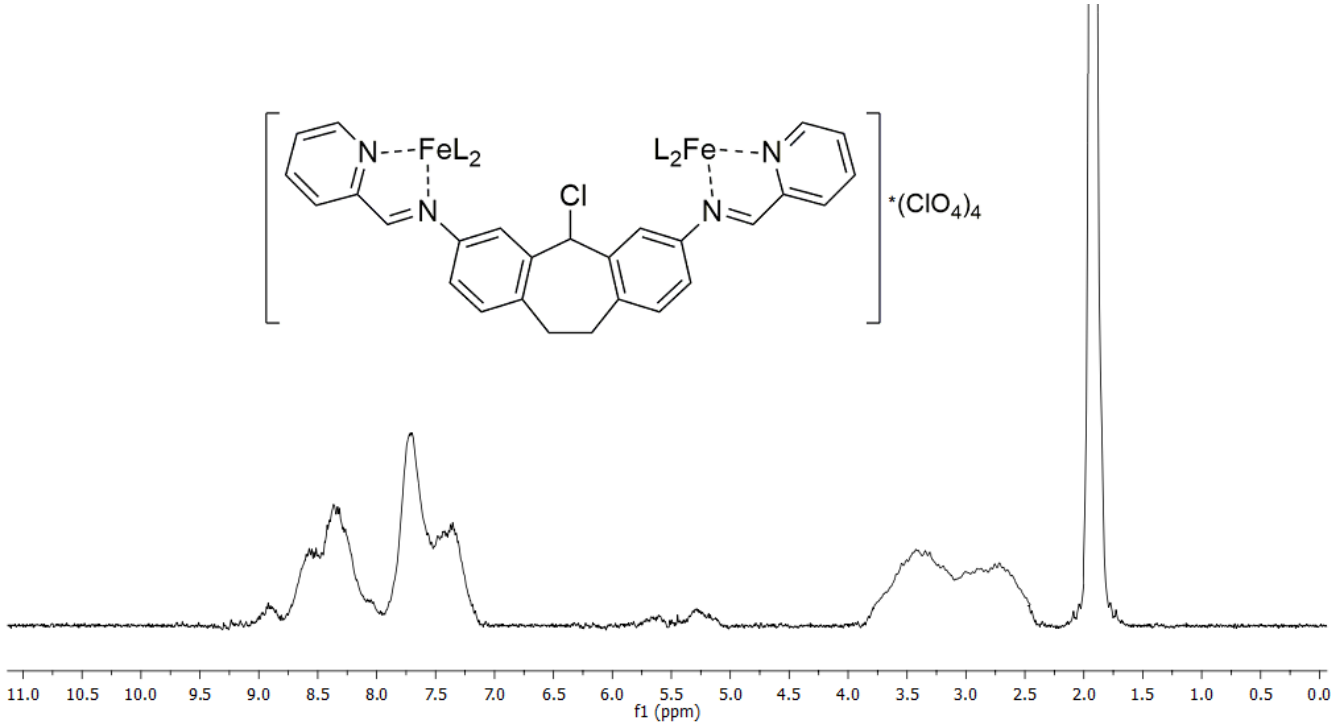

Figure S7. ^{1}H NMR spectrum of Aggregate **3** (CD_3CN, 600 MHz, 298 K).

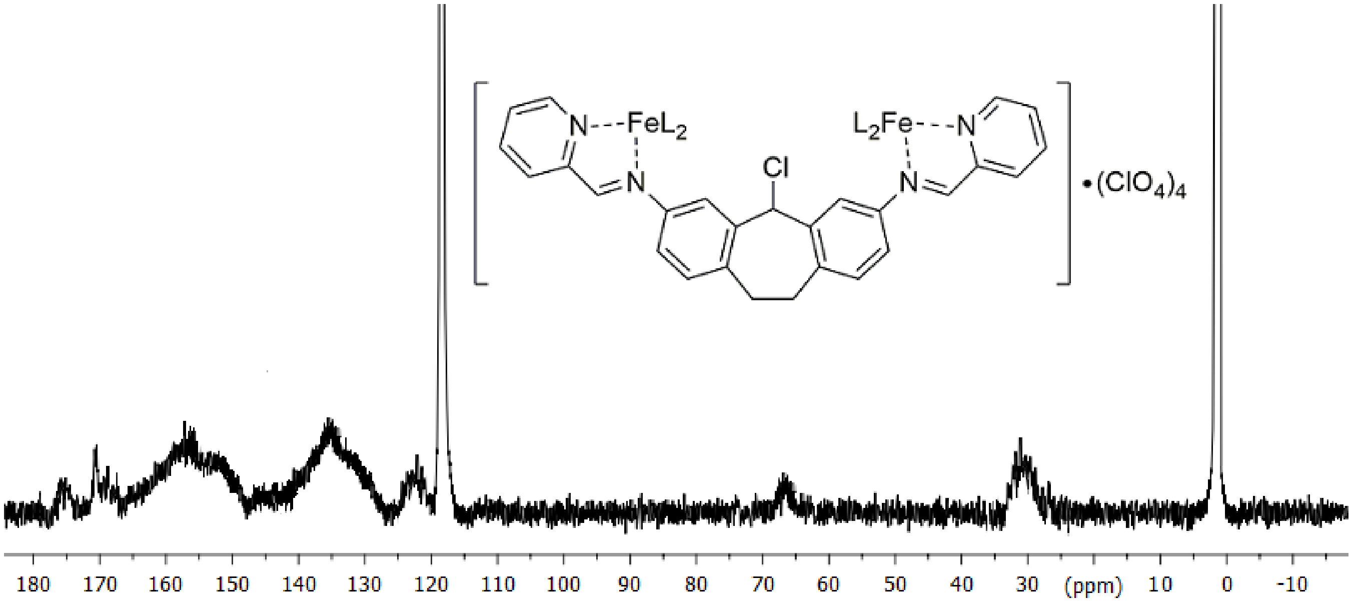

Figure S8. ^{13}C NMR spectrum of Aggregate **3** (CD_3CN, 150 MHz, 298 K).

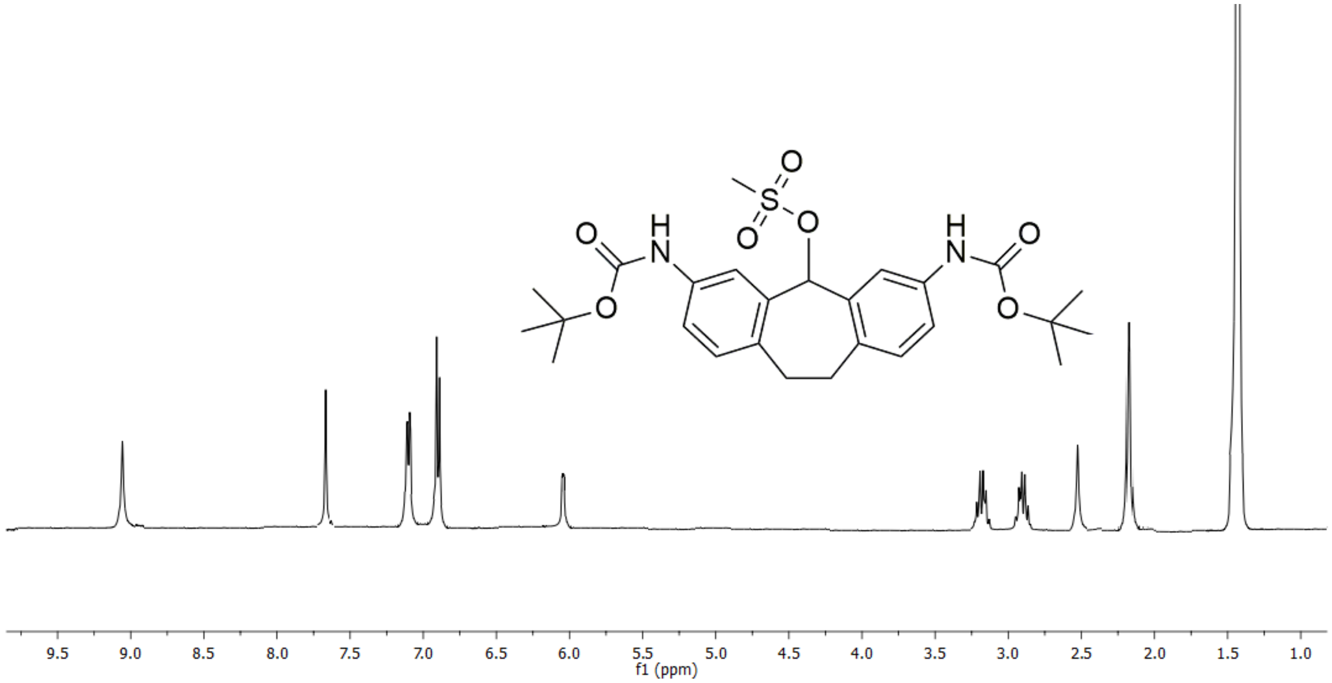

Figure S9. ^{1}H NMR spectrum of ***S*-3** (DMSO-d_6, 400 MHz, 298 K).

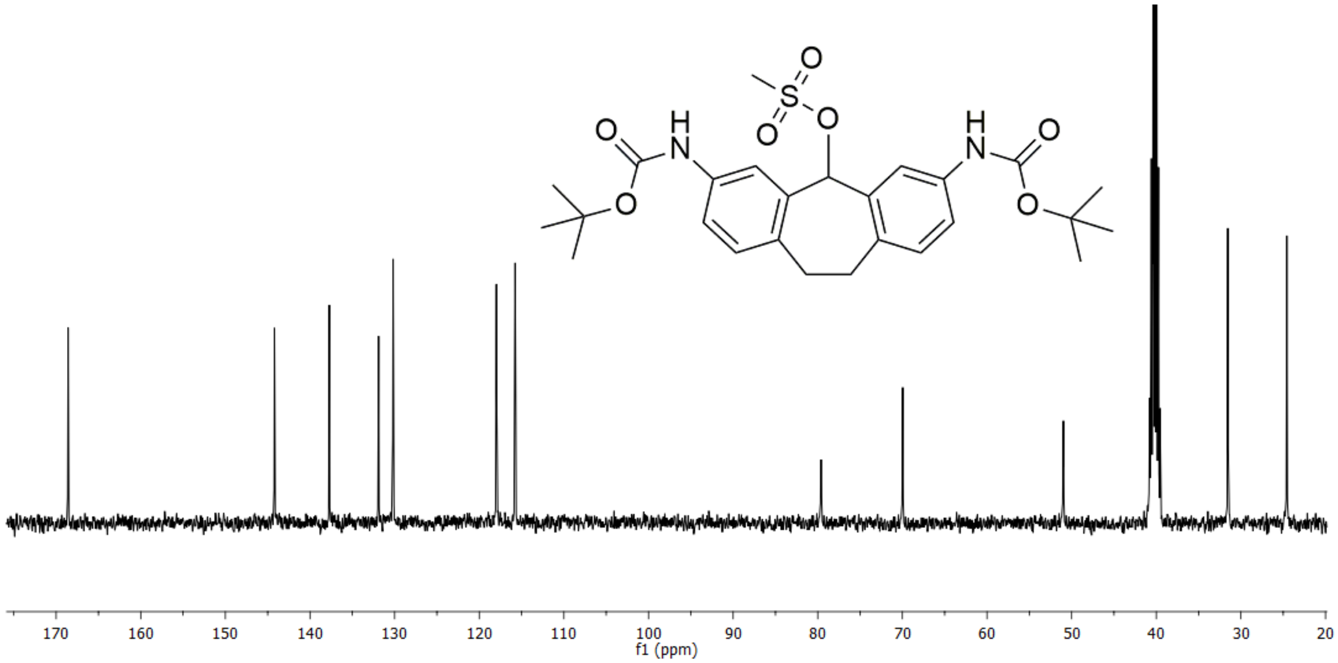

Figure S10. ^{13}C NMR spectrum of ***S*-3** (DMSO-d_6, 100 MHz, 298 K).

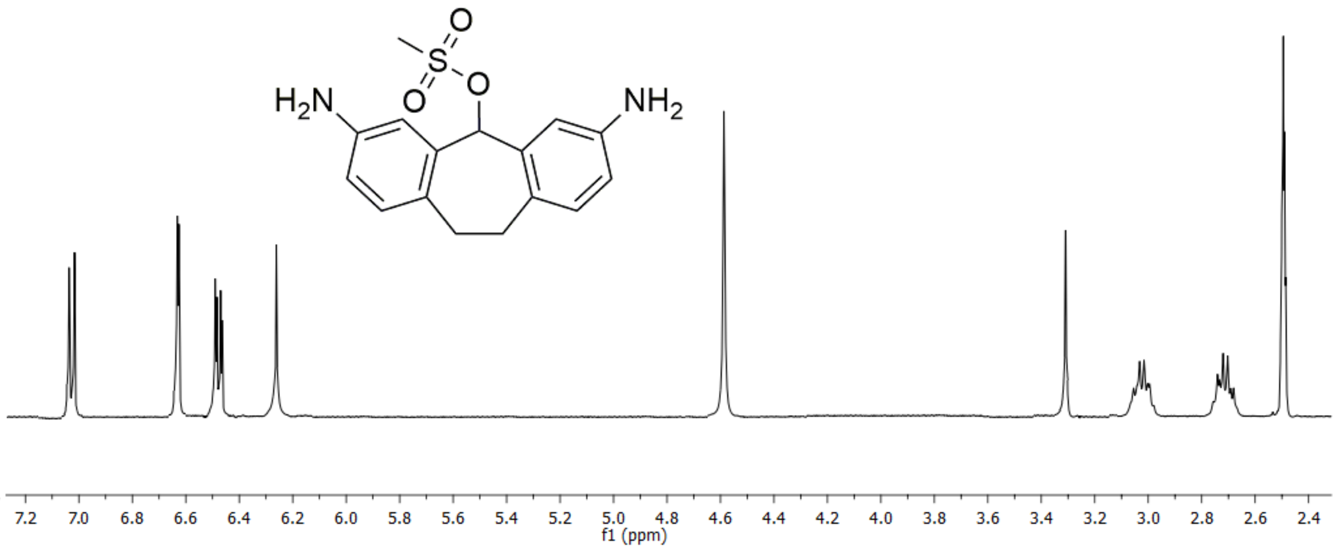

Figure S11. ^{1}H NMR spectrum of Dianiline **D** (DMSO-d_6, 400 MHz, 298 K).

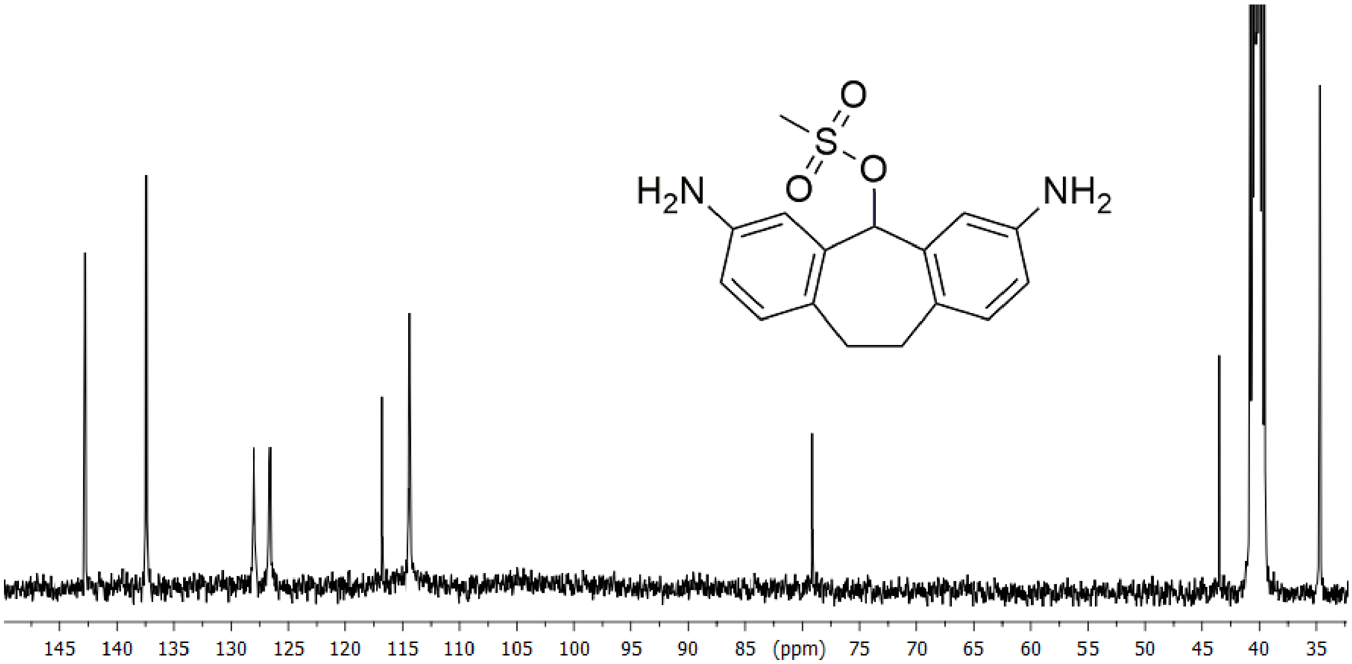

Figure S12. ^{13}C NMR spectrum of Dianiline **D** (DMSO-d_6, 100 MHz, 298 K).

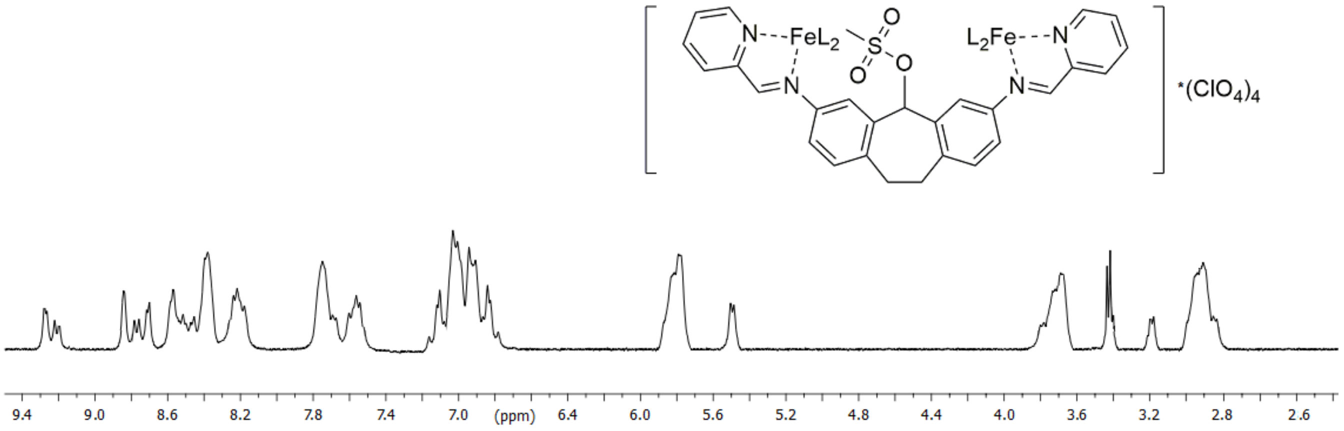

Figure S13. ^{1}H NMR spectrum of Mesylate cage **4** (CD_3CN, 600 MHz, 298 K).

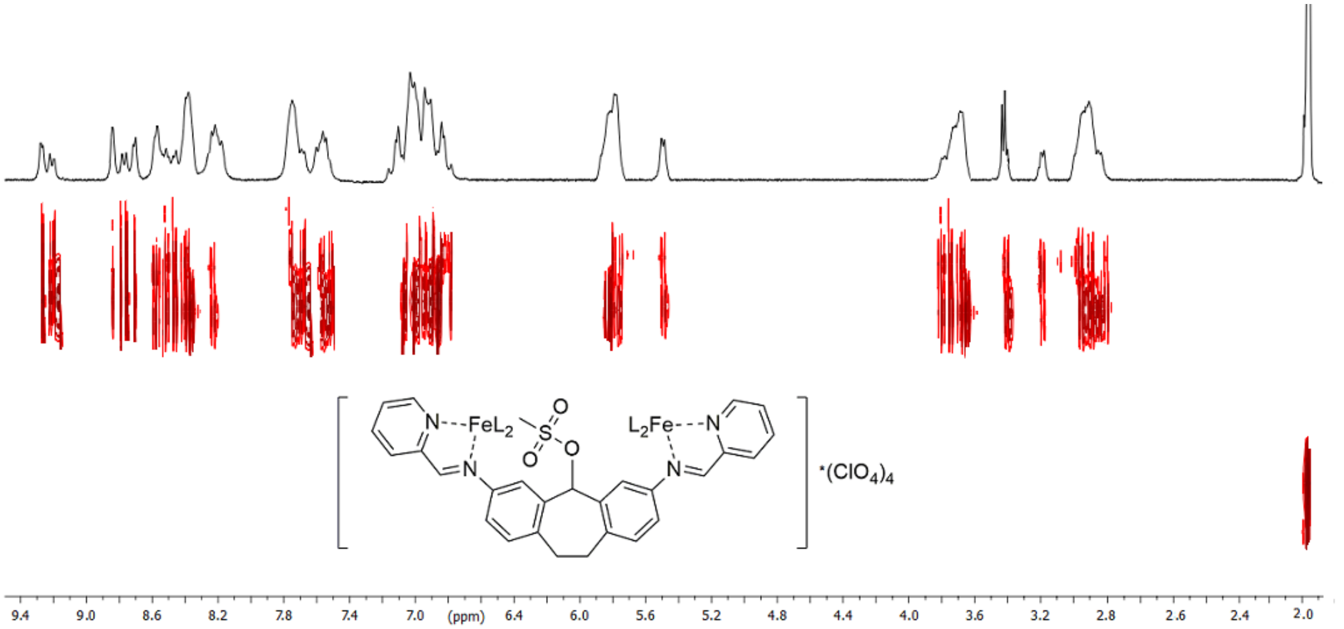

Figure S14. ^{1}H-DOSY NMR spectrum of Mesylate Cage **4** (CD_3CN, 600 MHz, 298 K, Δ = 100 ms, δ = 2.6 µs, Diffusion Coefficient = 2.04 x10^{-9} m^2/s for cage **4** vs. 4.97 x10^{-9} m^2/s for the solvent).

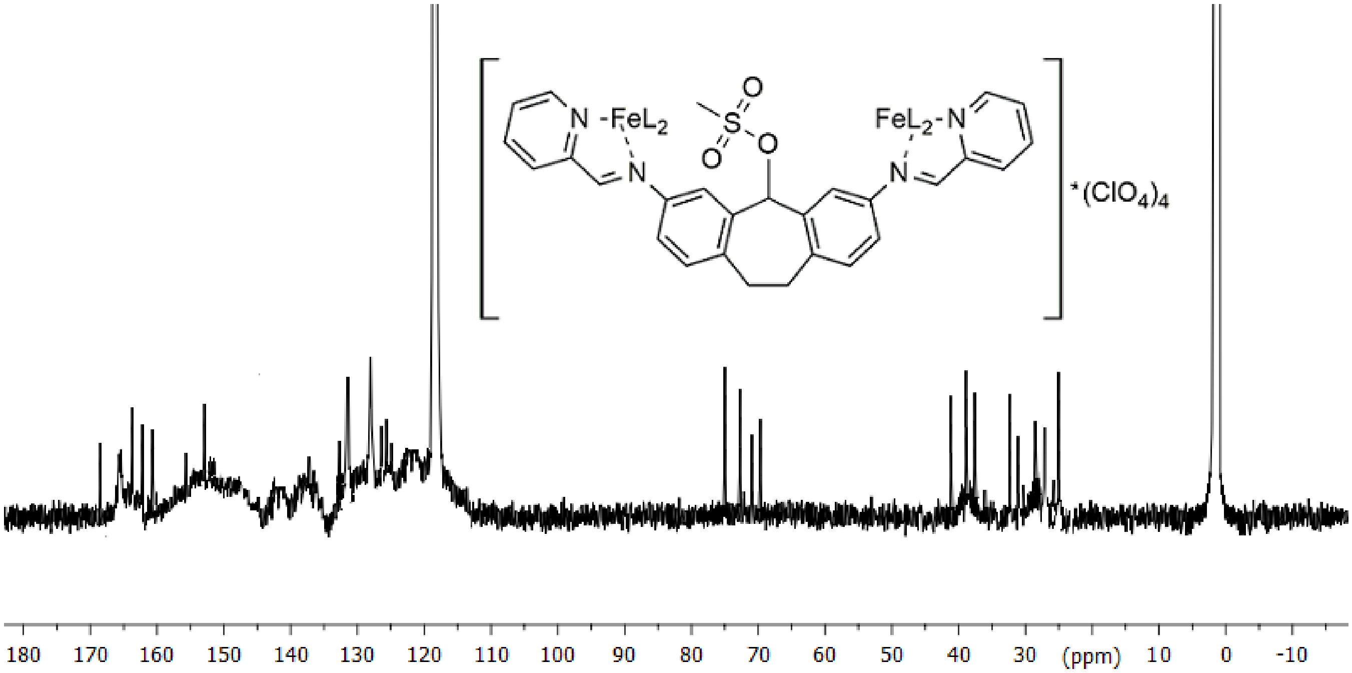

Figure S15. ^{13}C NMR spectrum of Mesylate cage **4** (CD_3CN, 150 MHz, 298 K).

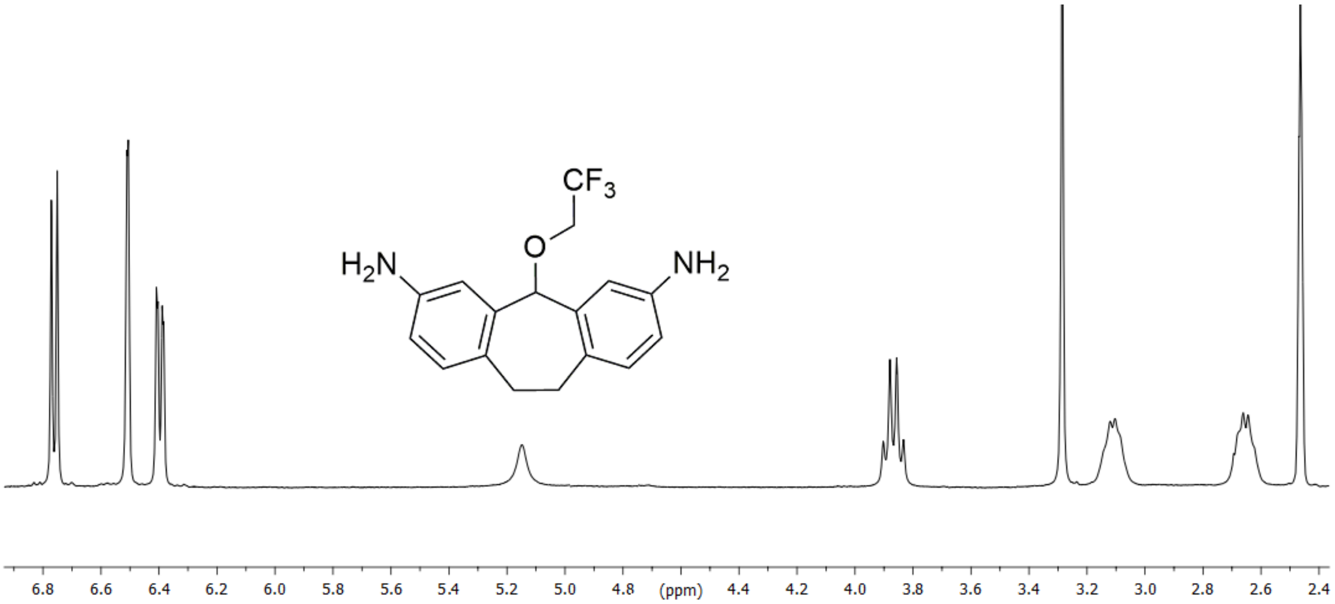

Figure S16. ^{1}H NMR spectrum of Dianiline **E** (DMSO, 400 MHz, 298 K).

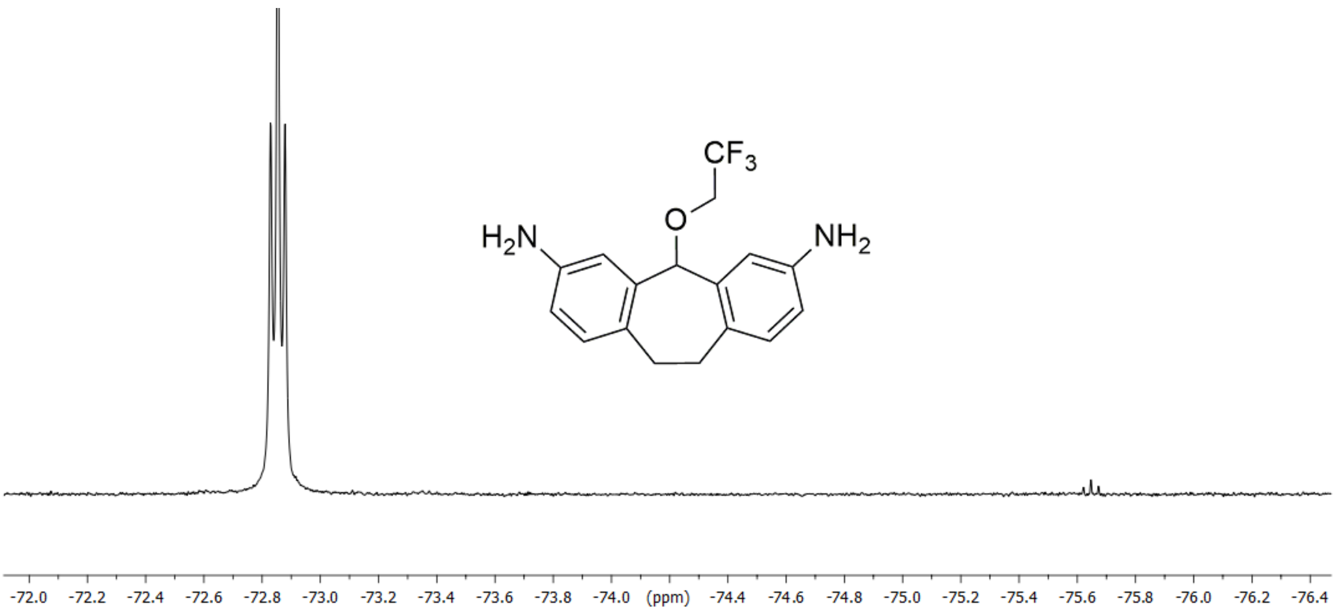

Figure S17. ^{19}F NMR spectrum of Dianiline **E** (DMSO-d_6, 400 MHz, 298 K).

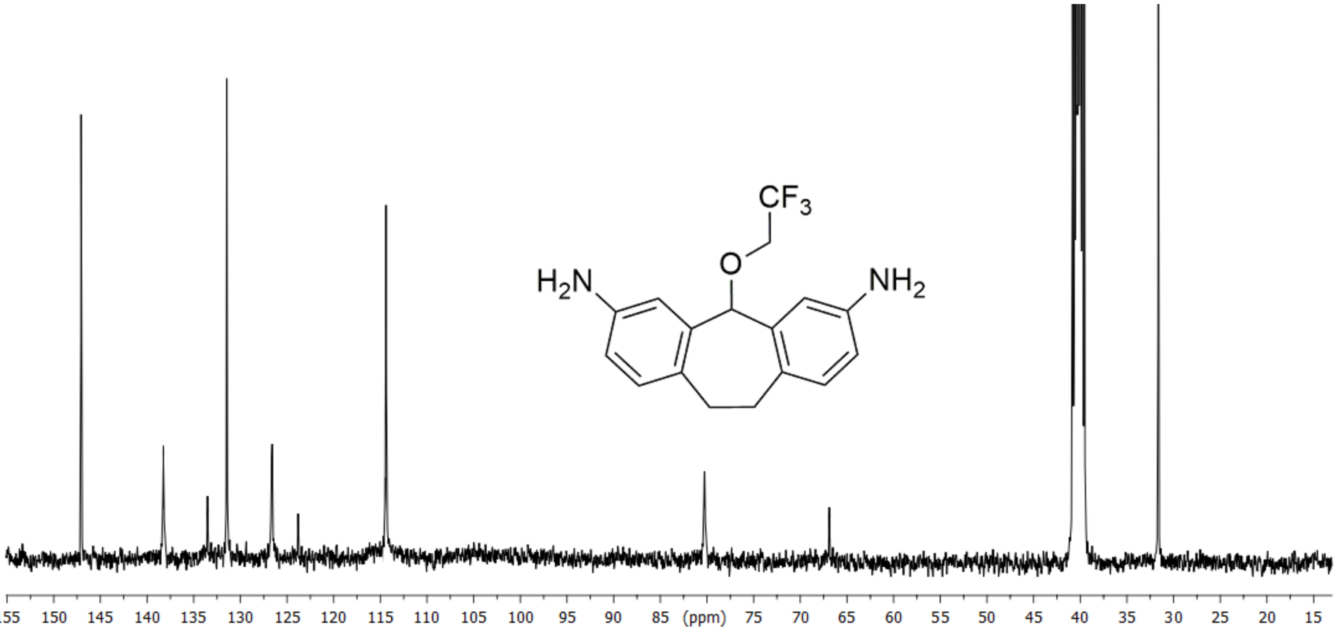

Figure S18. ^{13}C NMR spectrum of Dianiline **E** (DMSO-d_6, 100 MHz, 298 K).

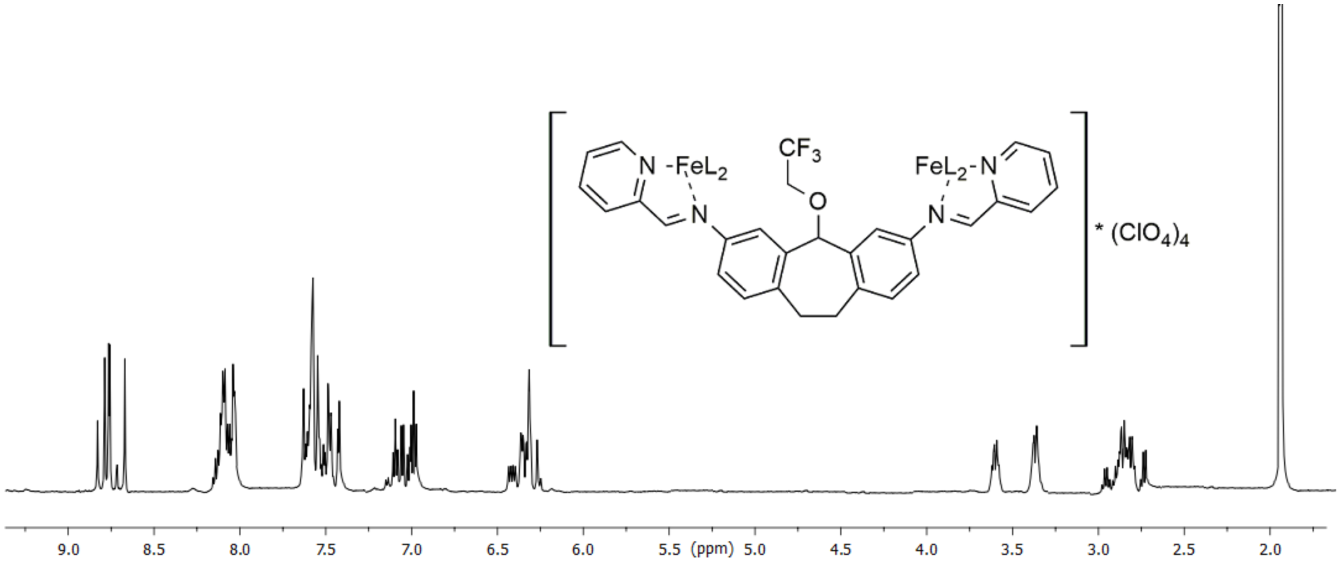

Figure S19. ^{1}H NMR spectrum of Trifluoroether Cage **5** (CD_3CN, 600 MHz, 298 K).

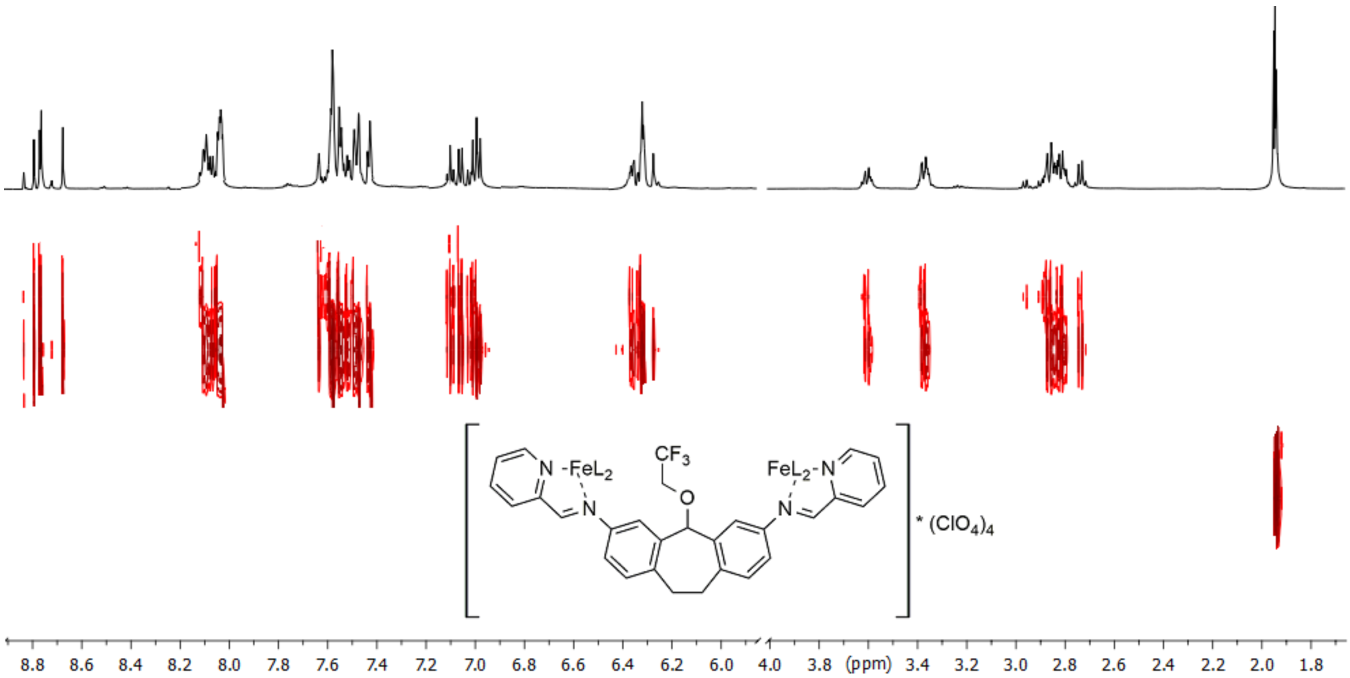

Figure S20. ^{1}H-DOSY NMR spectrum of Trifluoroether Cage **5** (CD_3CN, 600 MHz, 298 K, Δ = 100 ms, δ = 2.6 µs, Diffusion Coefficient = 1.14 x10^{-9} m^2/s for cage **5** vs. 4.43 x10^{-9} m^2/s for the solvent).

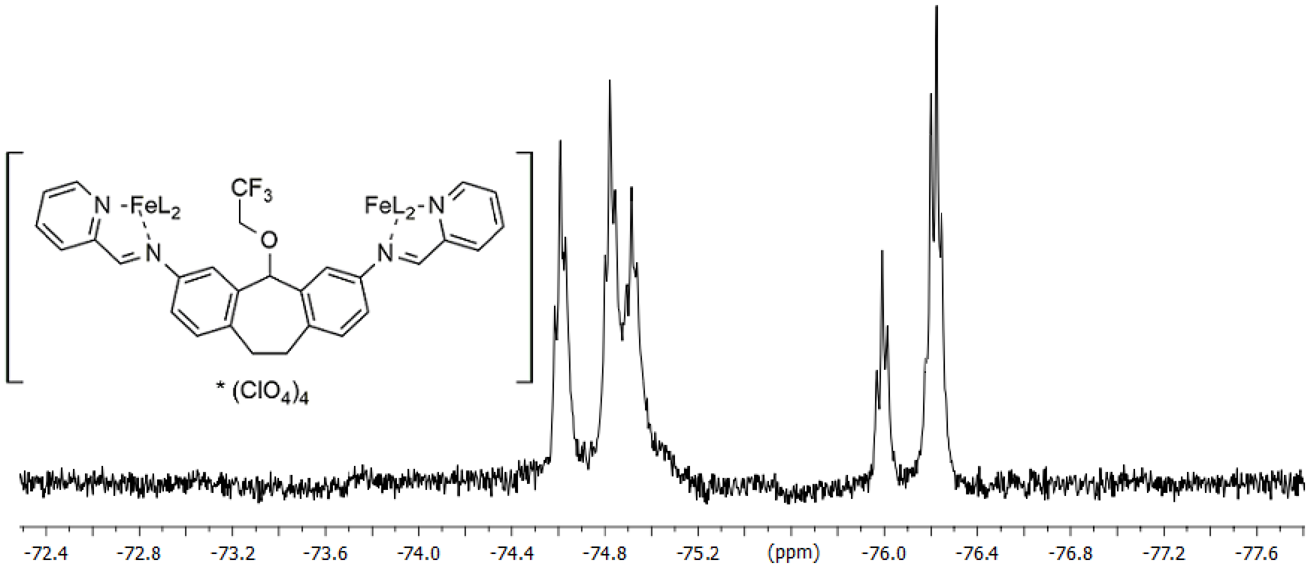

Figure S21. ^{19}F NMR spectrum of Trifluoroether Cage **5** (CD_3CN, 400 MHz, 298 K).

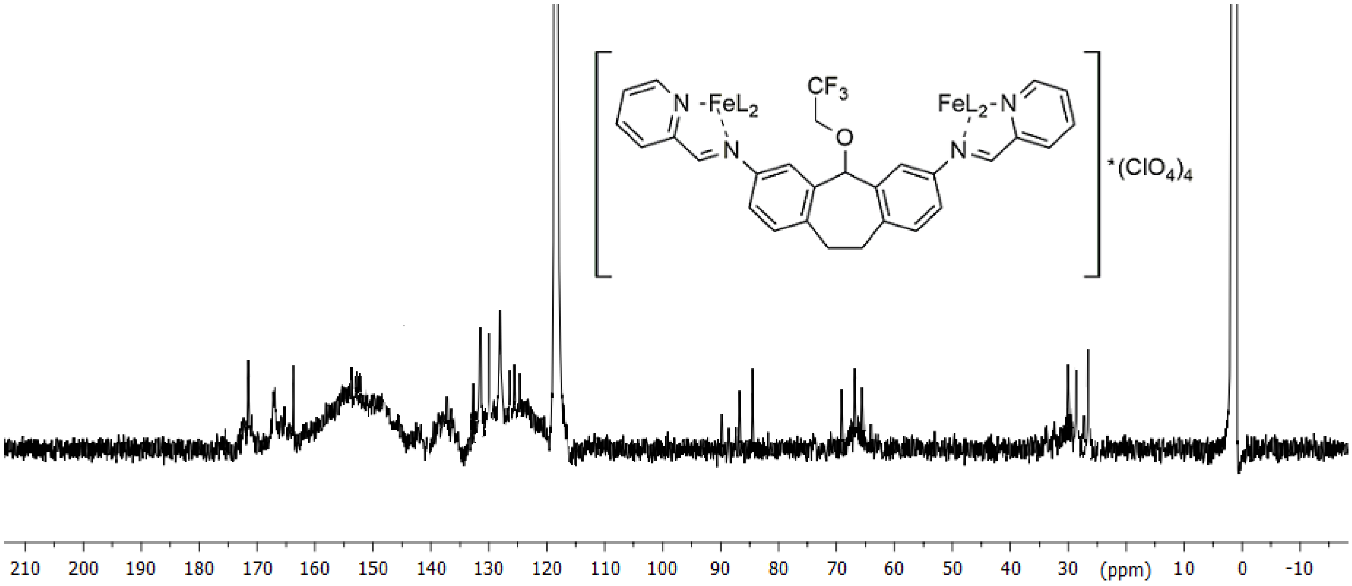

Figure S22. ^{13}C NMR spectrum of Trifluoroether Cage **5** (CD_3CN, 150 MHz, 298 K).

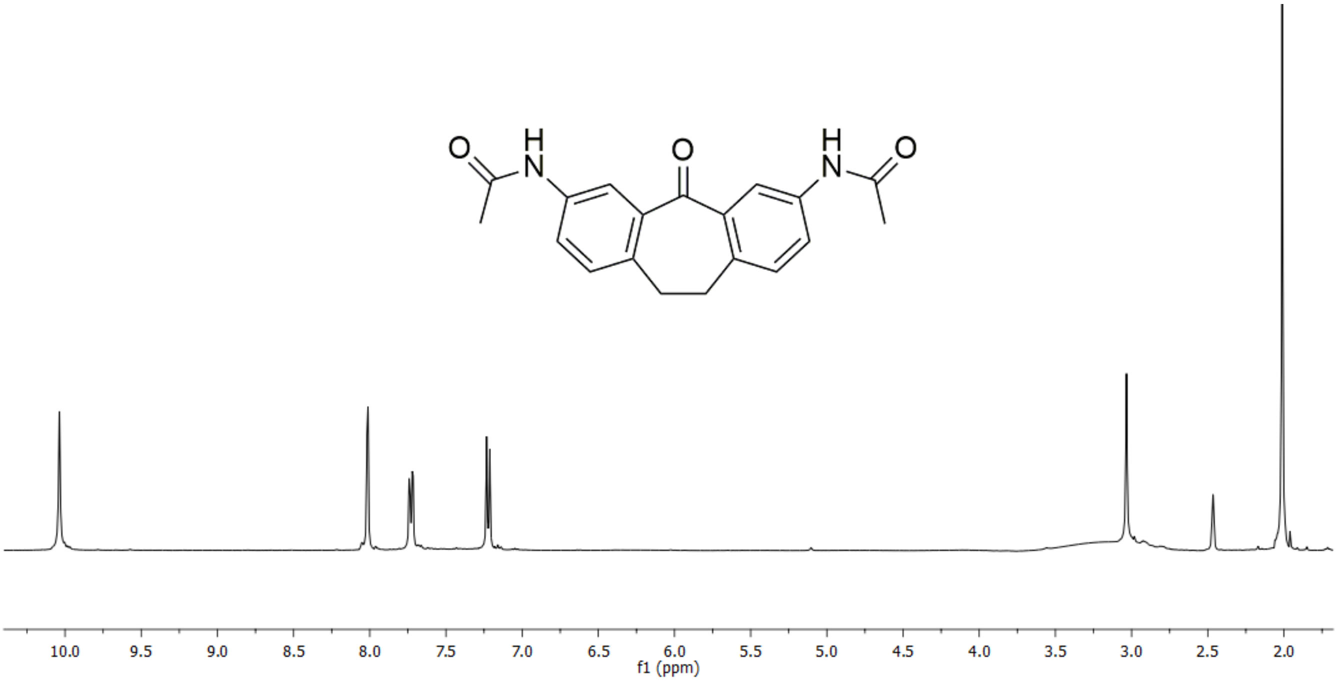

Figure S23. ^{1}H NMR spectrum of ***S*-3** (DMSO-d_6, 400 MHz, 298 K).

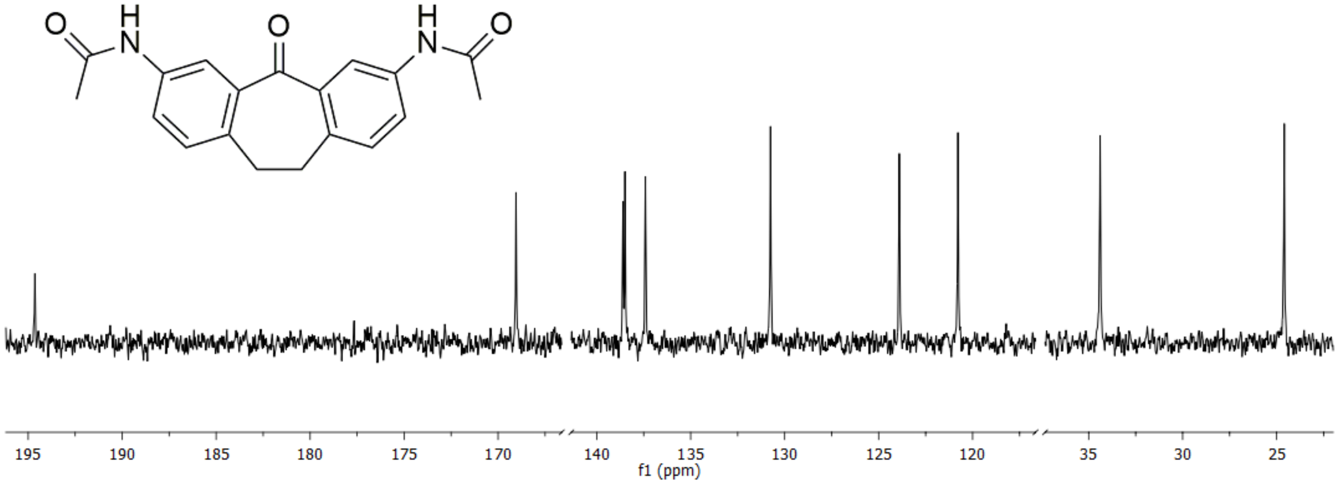

Figure S24. ^{13}C NMR spectrum of ***S*-3** (DMSO-d_6, 100 MHz, 298 K).

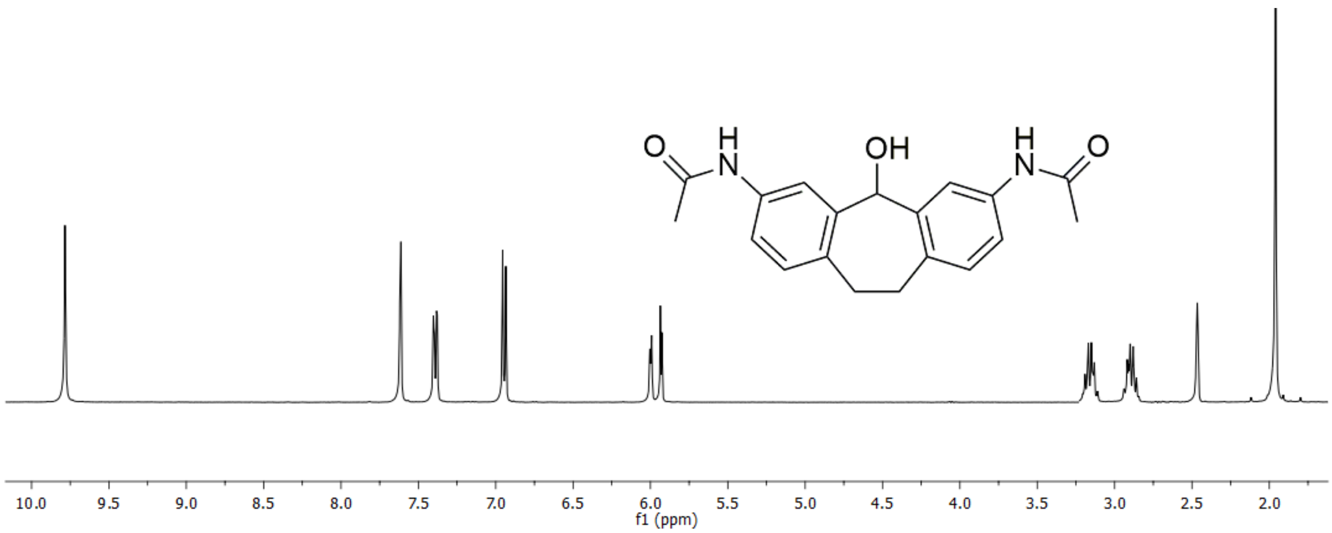

Figure S25. ^{1}H NMR spectrum of **7** (DMSO-d_6, 400 MHz, 298 K).

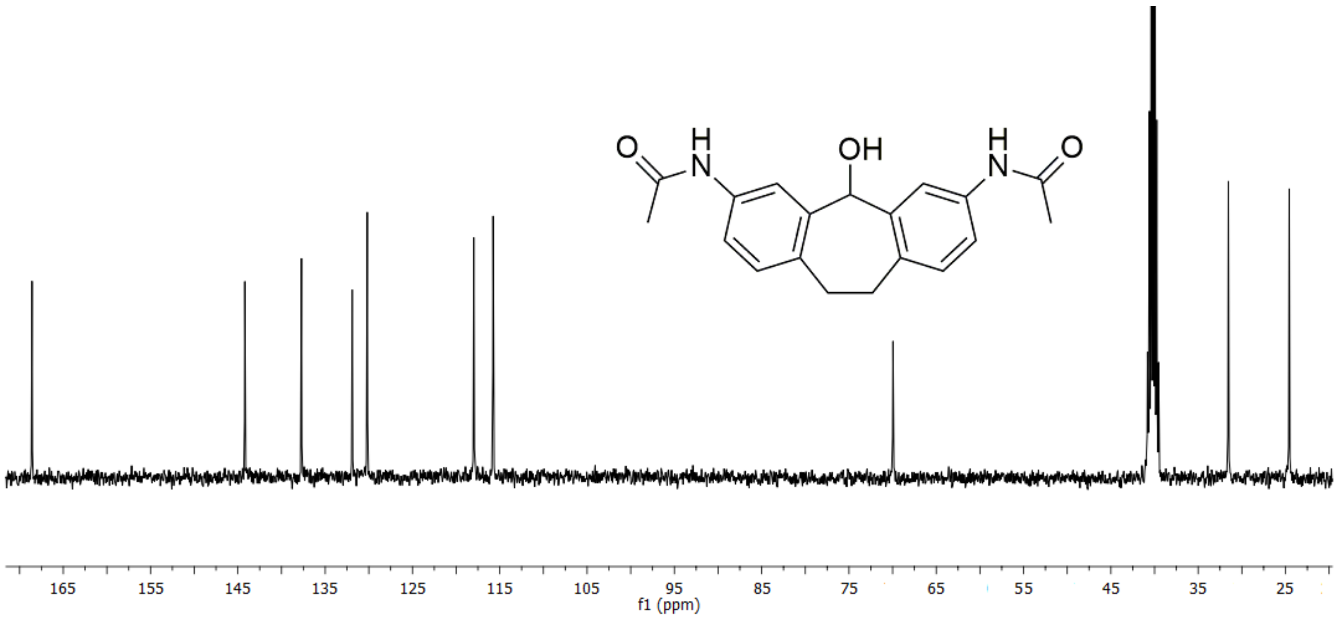

Figure S26. ^{13}C NMR spectrum of **7** (DMSO-d_6, 100 MHz, 298 K).

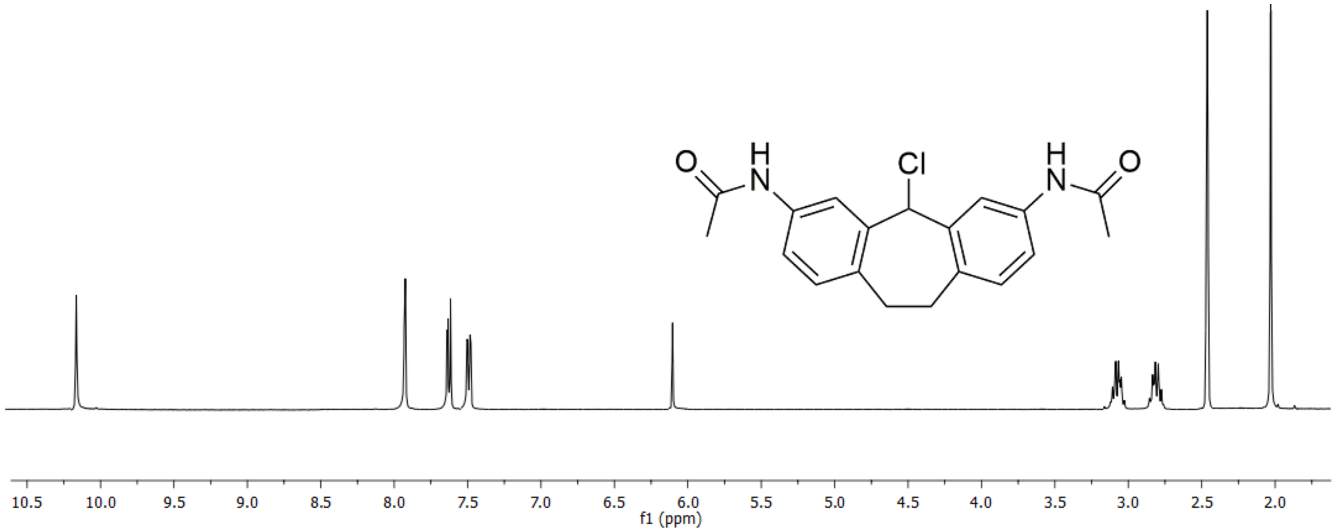

Figure S27. ^{1}H NMR spectrum of **6** (DMSO-d_6, 400 MHz, 298 K).

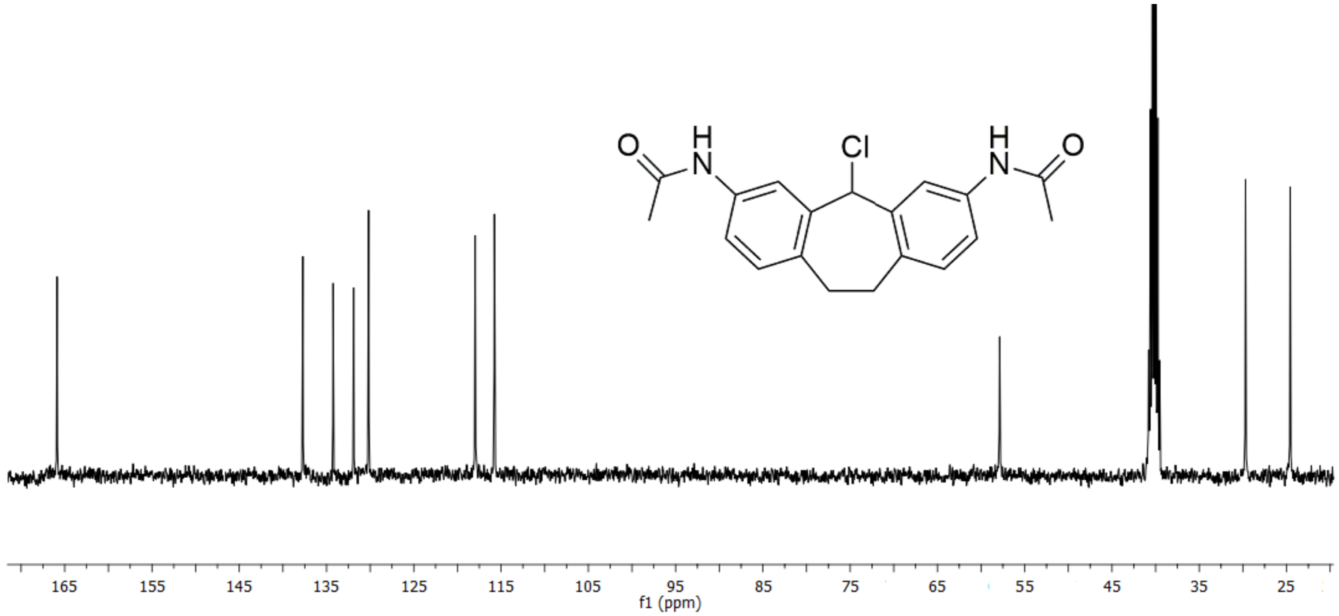

Figure S28. ^{13}C NMR spectrum of **6** (DMSO-d_6, 100 MHz, 298 K).

Mass Spectral Data

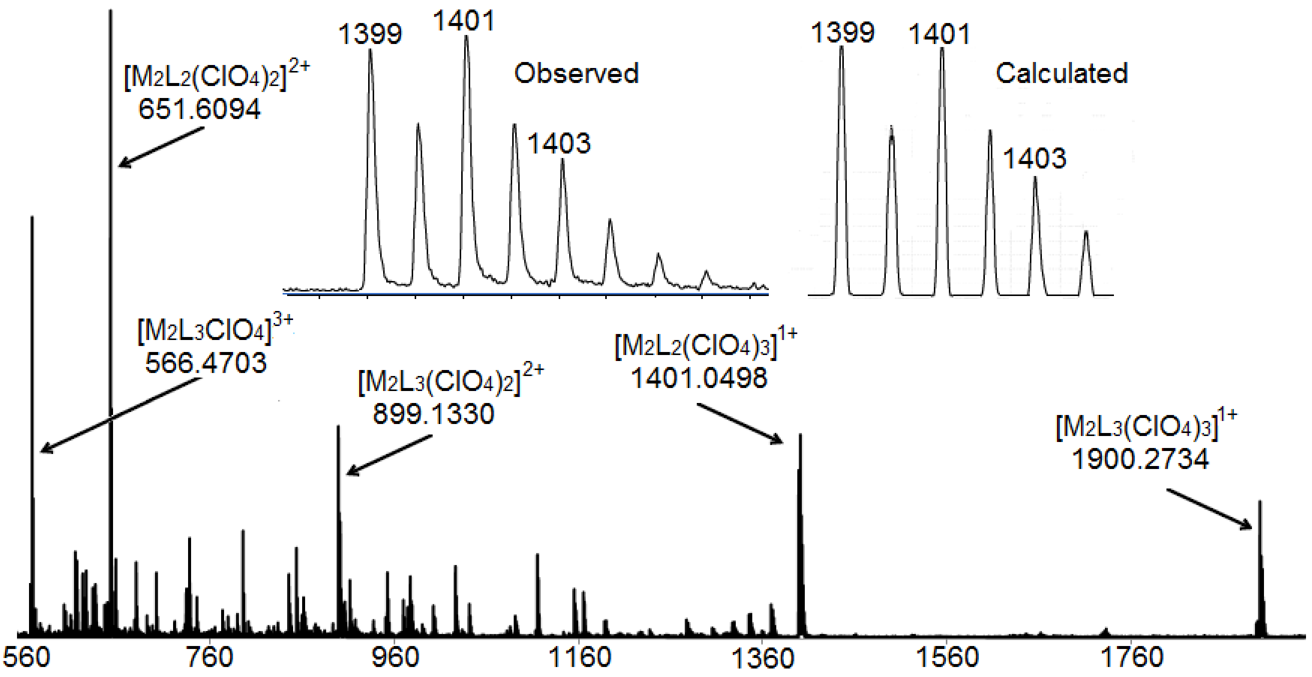

Figure S29. ESI-MS of Mesylate cage **4** (CH_3CN).

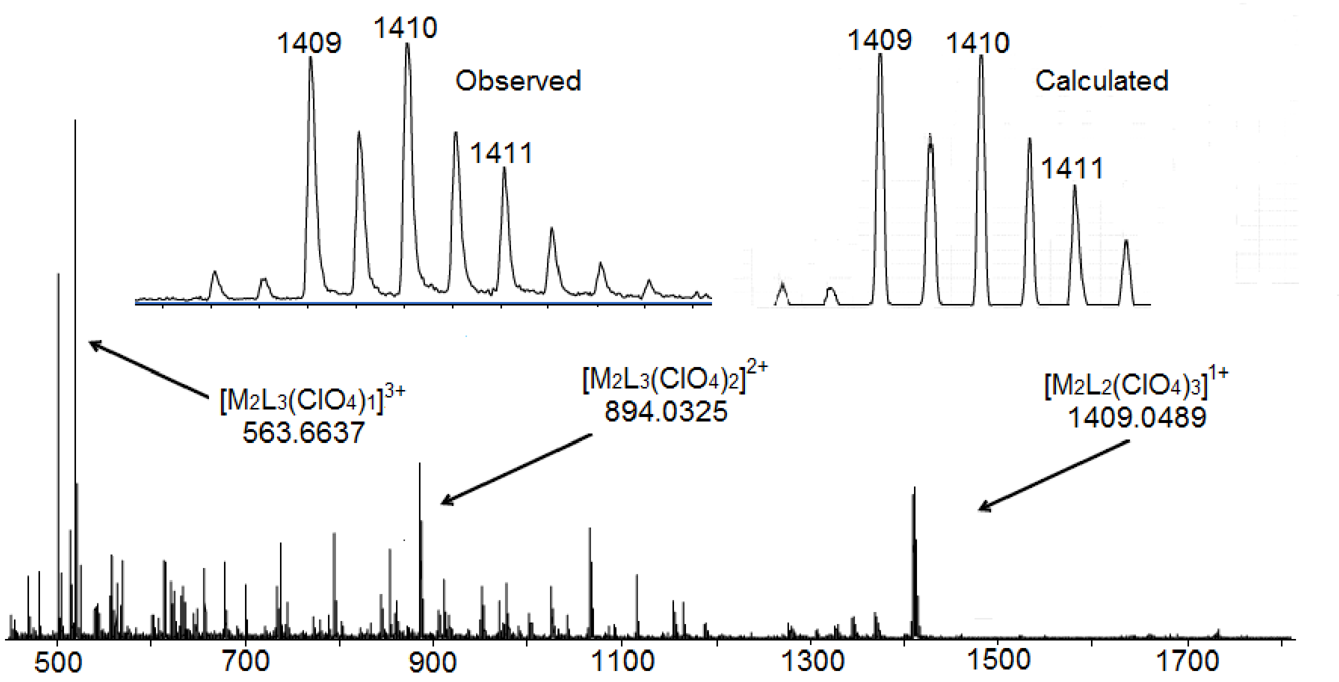

Figure S30. ESI-MS of Trifluoroether cage **5** (CH_3CN).

Cage Oxidation Experiments

General oxidation procedure: All mixing experiments were performed in an NMR tube. Aggregate **3** was formed *in situ* in an NMR tube: Dianiline **C** (10 mg, 0.039 mmol) was placed in an NMR tube along with 2 molar equivalents of 2-formylpyridine (7.4 µL, 0.077 mmol) and 0.66 molar equivalents of Iron (II) perchlorate (0.026 mmol, 8.3 mg). The tube contents were quickly mixed in an ultrasonication bath and allowed to heat at 45°C for 18 h to form the aggregate. Premade cages **2**, **4** and **5** were synthesized and isolated as described above, and the dry solid weighed into an NMR tube (0.039 mmol). One molar equivalent of silver perchlorate relative to chloride ligand (0.039 mmol, 8 mg) and 1 equivalent water (0.039 mmol, 5 µl of a 7.8 M H_2O solution in CD_3CN). The tube contents were quickly mixed and heated at 45 or 80°C. The NMR spectra of the reaction mixture were taken at regular intervals to monitor oxidation progress.

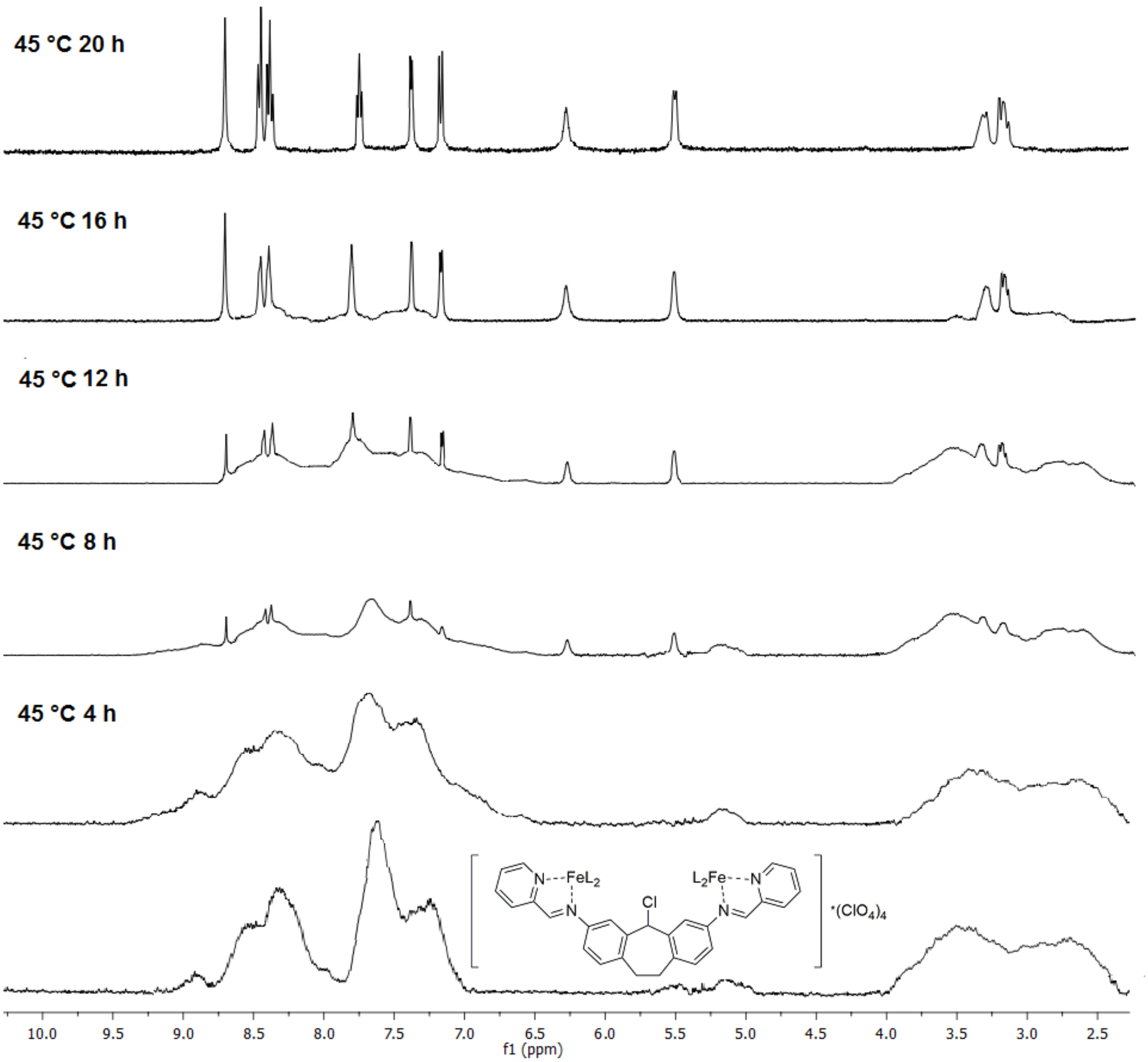

Figure S31. ^{1}H NMR spectrum of oxidation products obtained after aggregate **3** was heated at 45 °C for 20 h with 1 eq. silver perchlorate and water (CD_3CN, 400 MHz, 298 K). The aggregate was cleanly converted to suberone cage **1** within 20 h in acetonitrile or DMSO solvent.

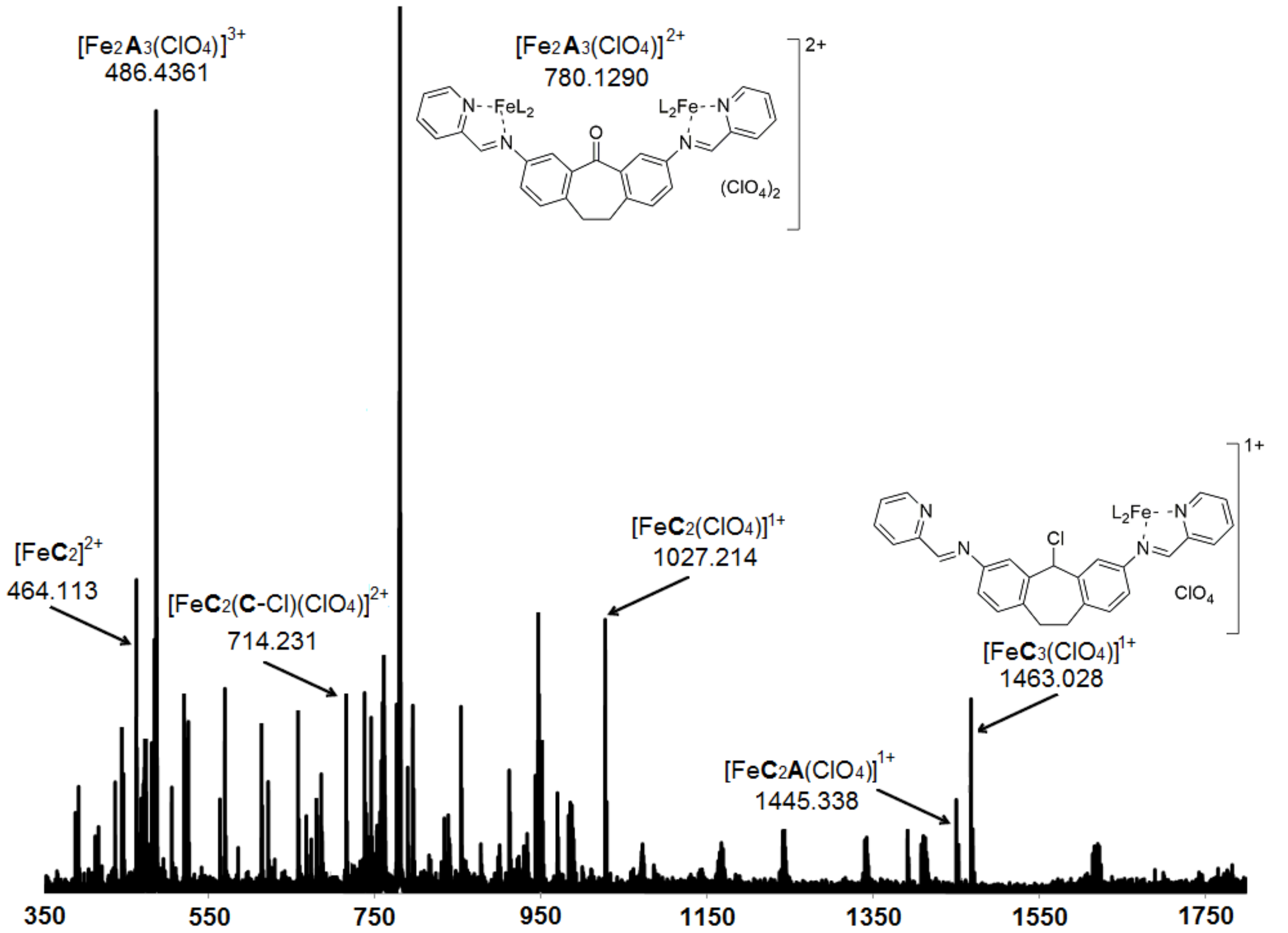

Figure S32. ESI-MS of reaction mixture after aggregate **3** was heated at 45 °C with one equivalent of silver perchlorate and water for 12 h (CD_3CN). Peaks corresponding to ketone M_2L_3 products are clearly present in addition to chloride ML_3 and ML_2 starting material fragments. One "heterocomplex" fragment was found containing one ketone iminopyridine ligand, two chloride iminopyridine ligands, one iron atom and one perchlorate anion.

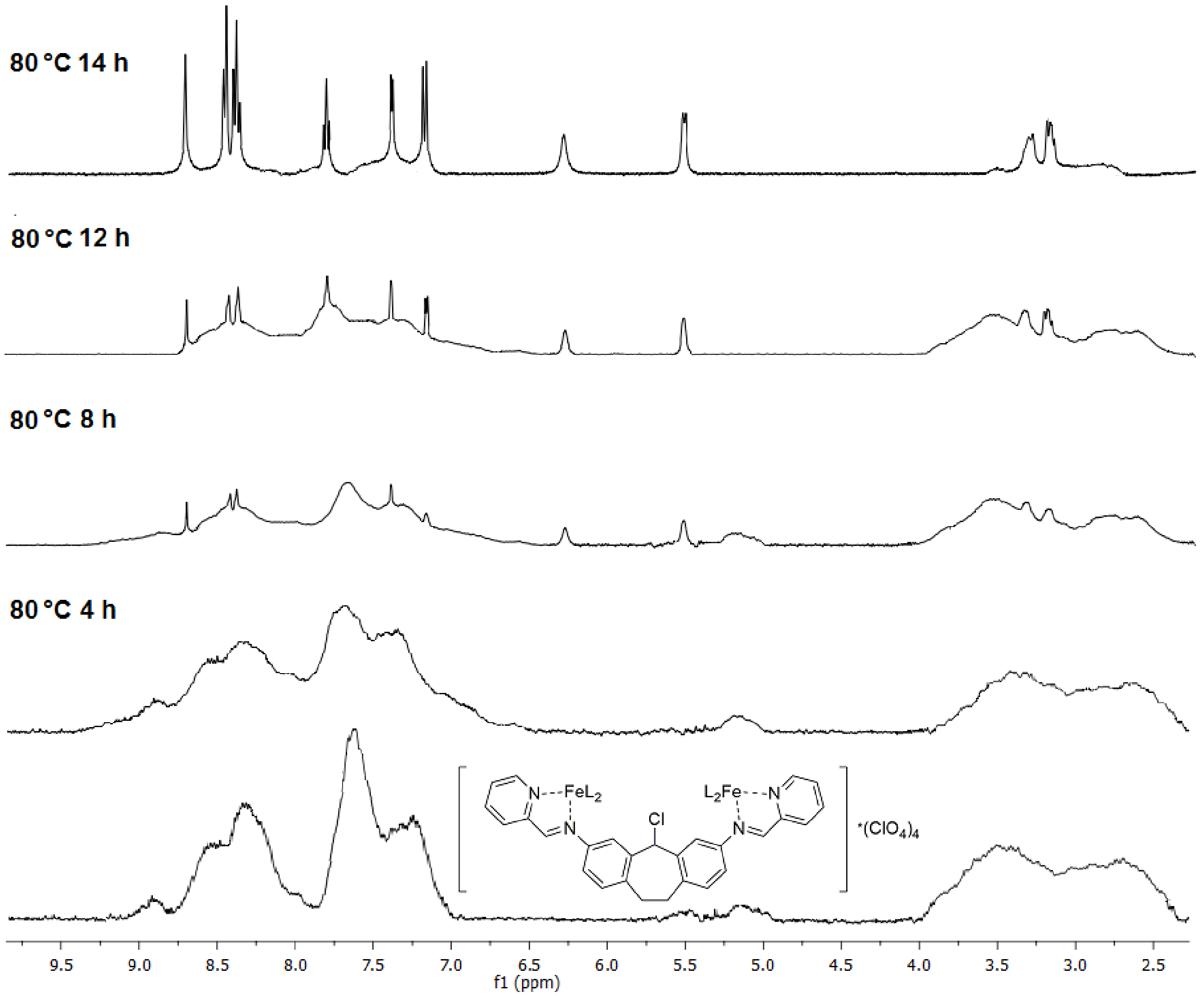

Figure S33. ^{1}H NMR spectrum of oxidation products obtained after aggregate **3** was heated at 80 °C for 14 h with 1 eq. silver perchlorate and water (CD_3CN, 400 MHz, 298 K). The aggregate was cleanly converted to suberone cage **1** within 14 h. Peak broadening is exhibited in the final product likely due to thermal decomposition.

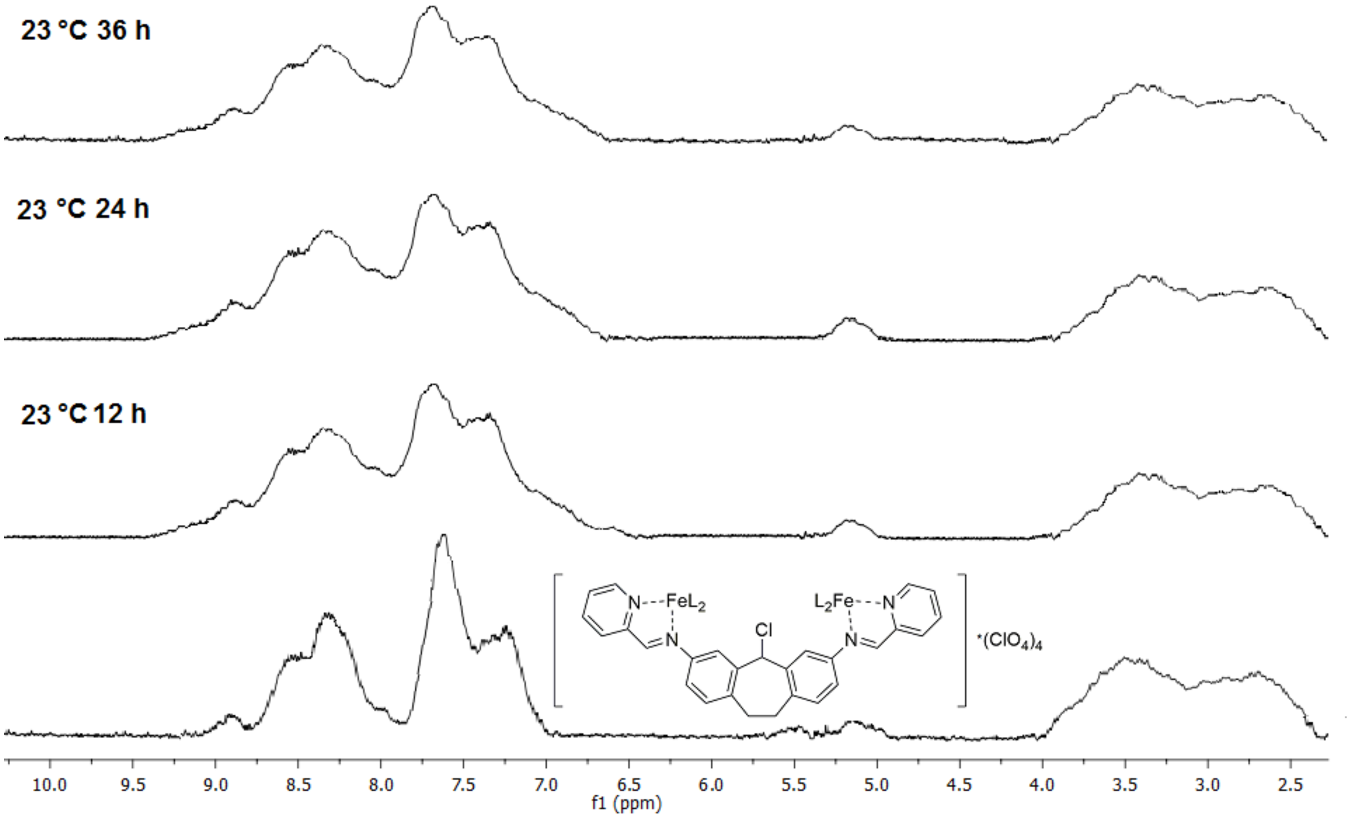

Figure S34. ^{1}H NMR spectrum of oxidation products obtained after aggregate **3** was heated at room temperature for 36 h with 1 eq. silver perchlorate and water (CD_3CN, 400 MHz, 298 K). No substantive changes were observed in the spectrum of the cage over time.

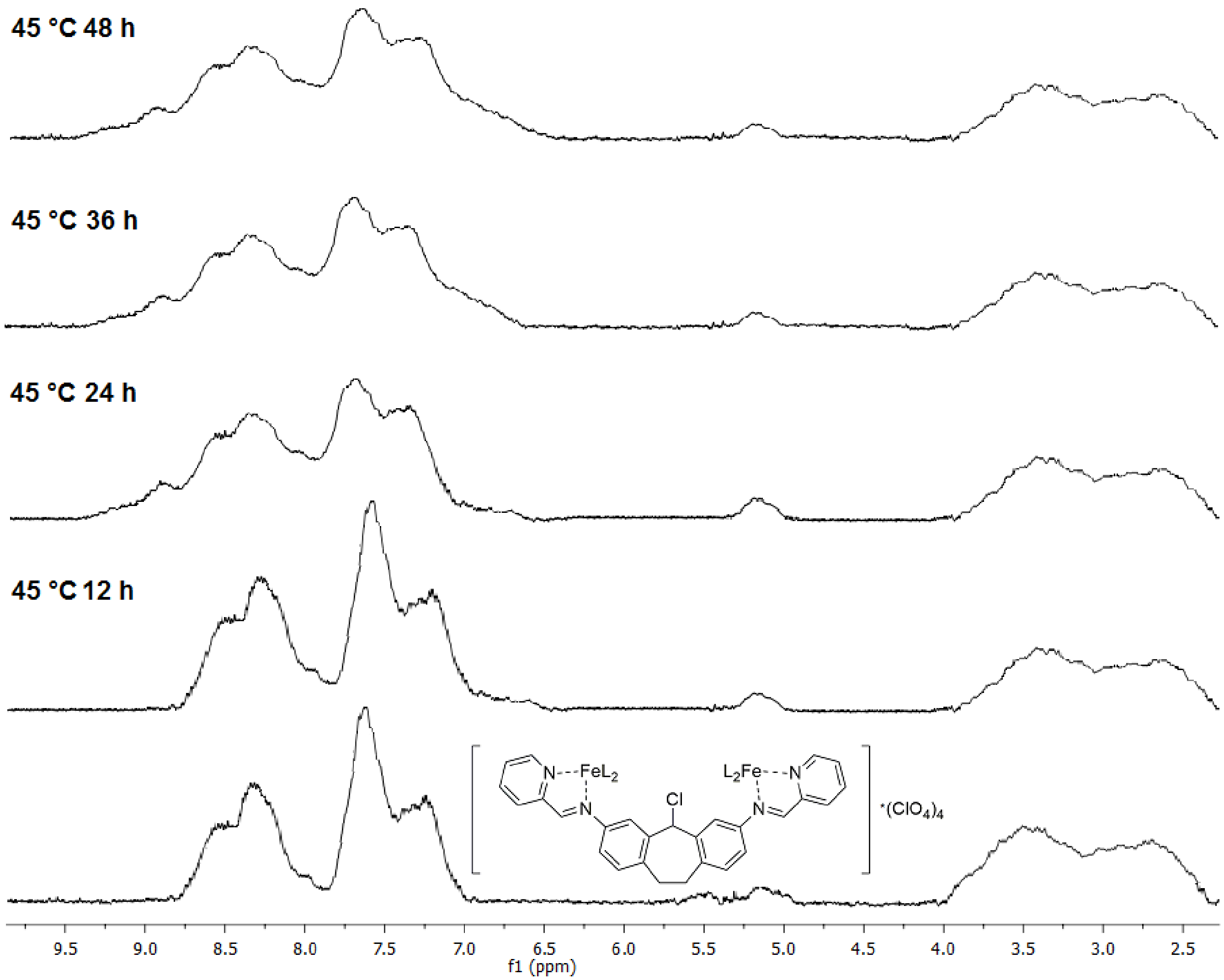

Figure S35. ^{1}H NMR spectrum of oxidation products obtained after aggregate **3** was heated at 45 °C for 48 h with 1 eq. silver perchlorate and water in the absence of O_2 (CD_3CN, 400 MHz, 298 K). No substantive changes were observed in the spectrum of the cage over time.

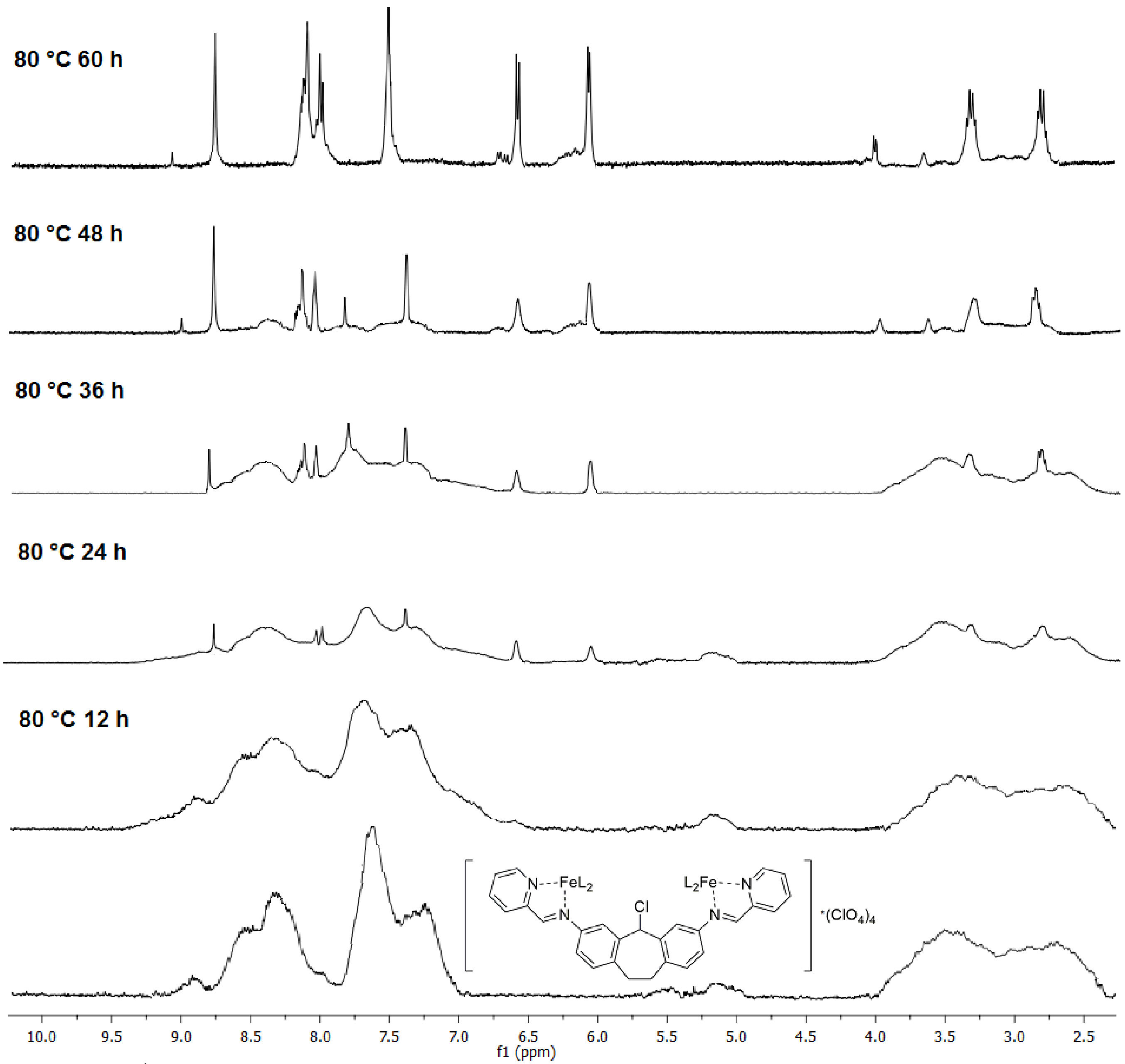

Figure S36. ^{1}H NMR spectrum of oxidation products obtained after aggregate **3** was heated at 80 °C for 60 h with 1 eq. silver perchlorate and water in the absence of O_2 (CD_3CN, 400 MHz, 298 K). Chloride aggregate **3** proceeds to the alcohol cage **2** with some thermal decomposition.

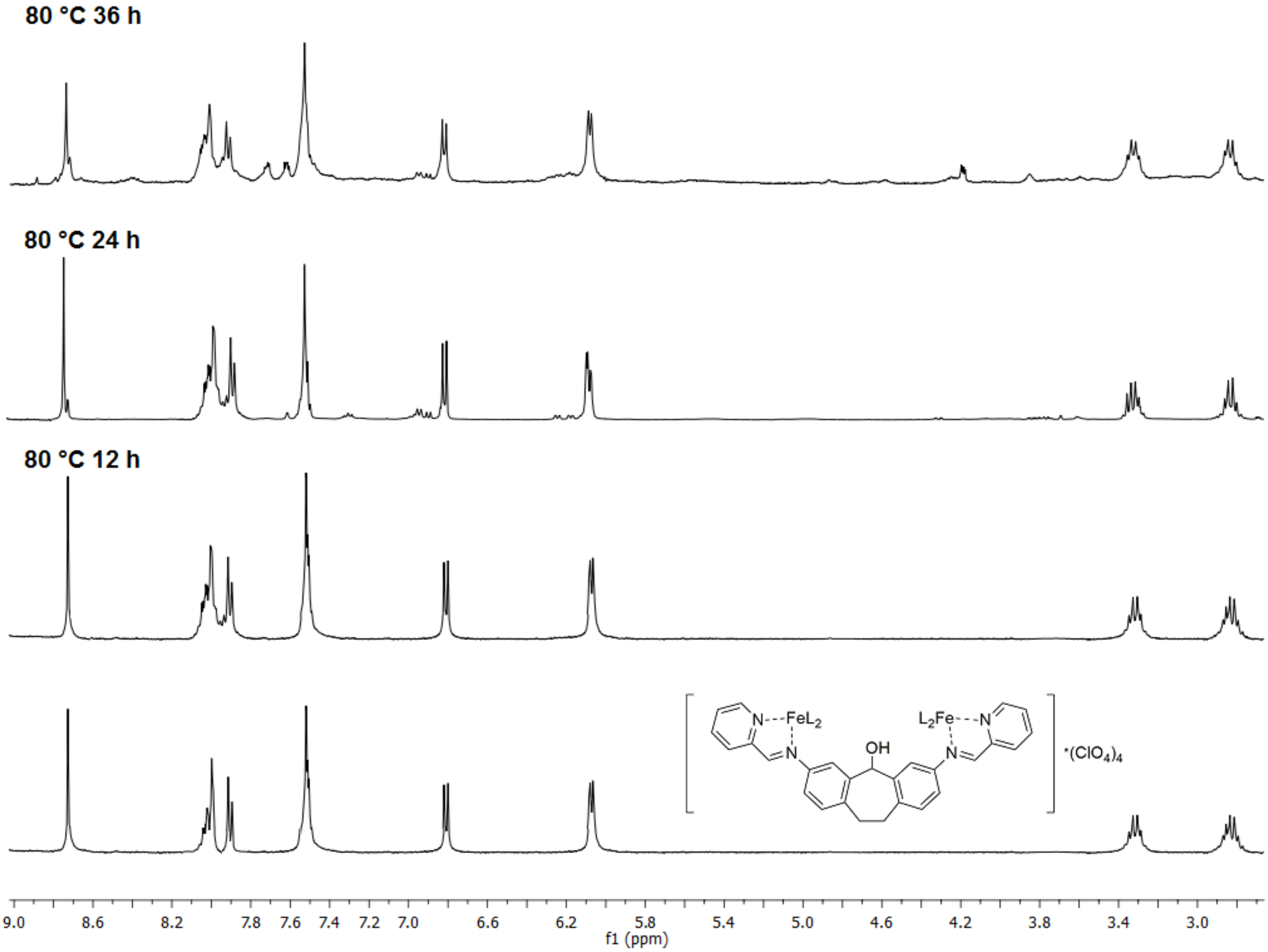

Figure S37. ^{1}H NMR spectrum of oxidation products obtained after suberol cage **2** was heated at 80 °C for 36 h with 1 eq. silver perchlorate and water (CD_3CN, 400 MHz, 298 K). No substantive changes were observed in the spectrum of the cage over time at 45 °C or 80 °C.

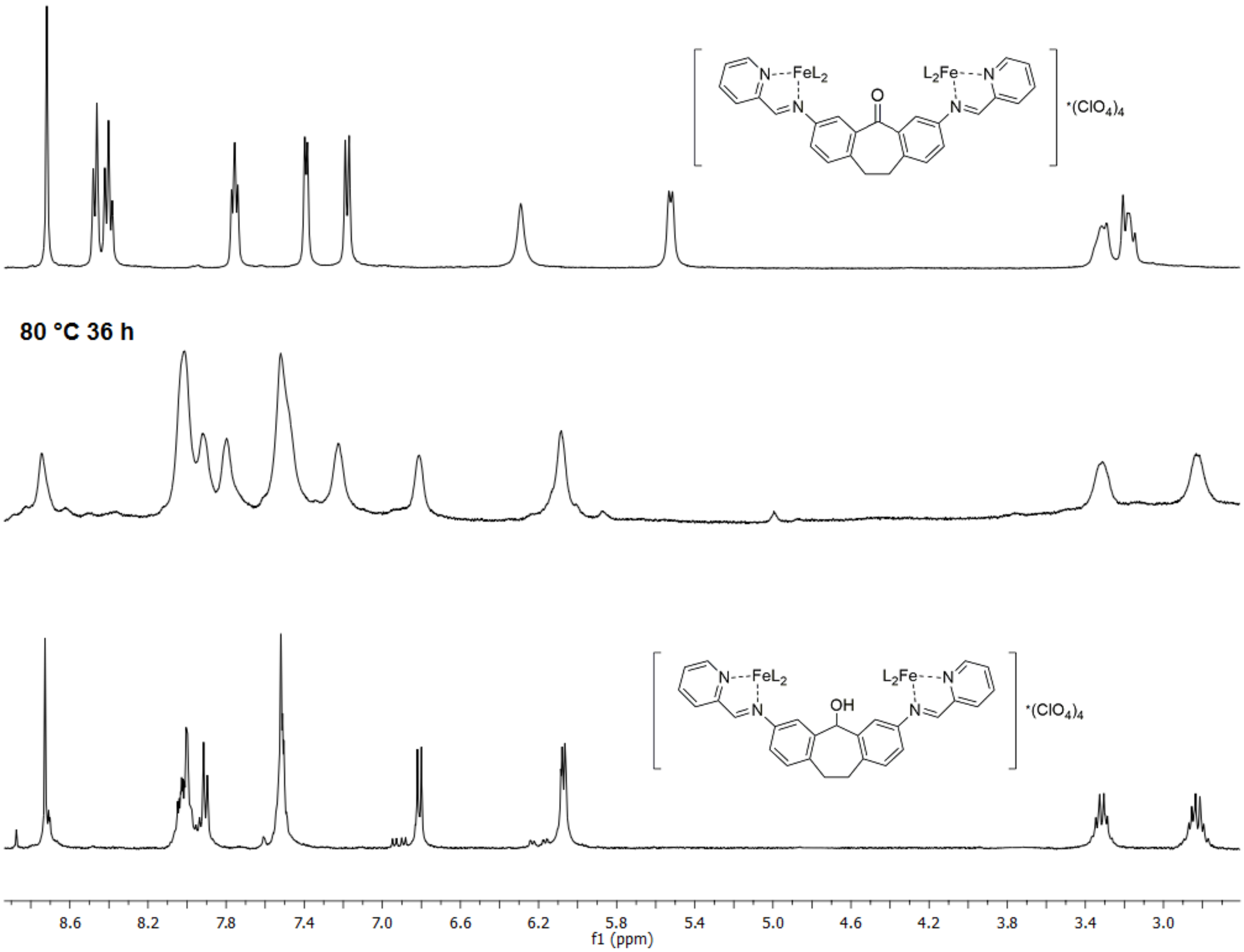

Figure S38. ^{1}H NMR spectrum of oxidation products obtained after suberol cage **2** was heated at 80 °C with 1 eq. Dess Martin Periodinane for 36 h (CD_3CN, 400 MHz, 298 K). Peak broadening did occur, however no trace of suberone cage **1** was formed.

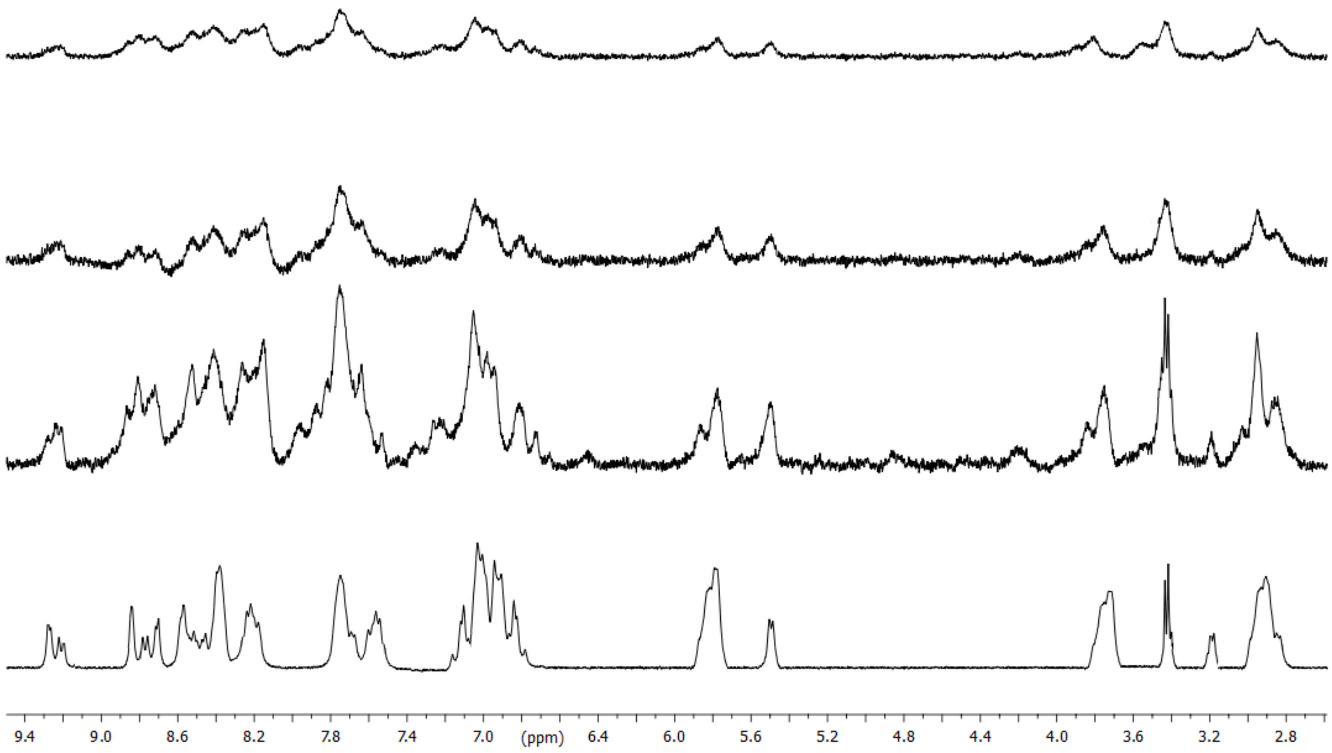

Figure S39. ^{1}H NMR spectrum of oxidation products obtained after mesylate cage **4** was heated at 80 °C for 36 h with 1 eq. silver perchlorate and water (CD_3CN, 600 MHz, 298 K). No substantive changes were observed in the spectrum of the cage over time at 45 °C or 80 °C.

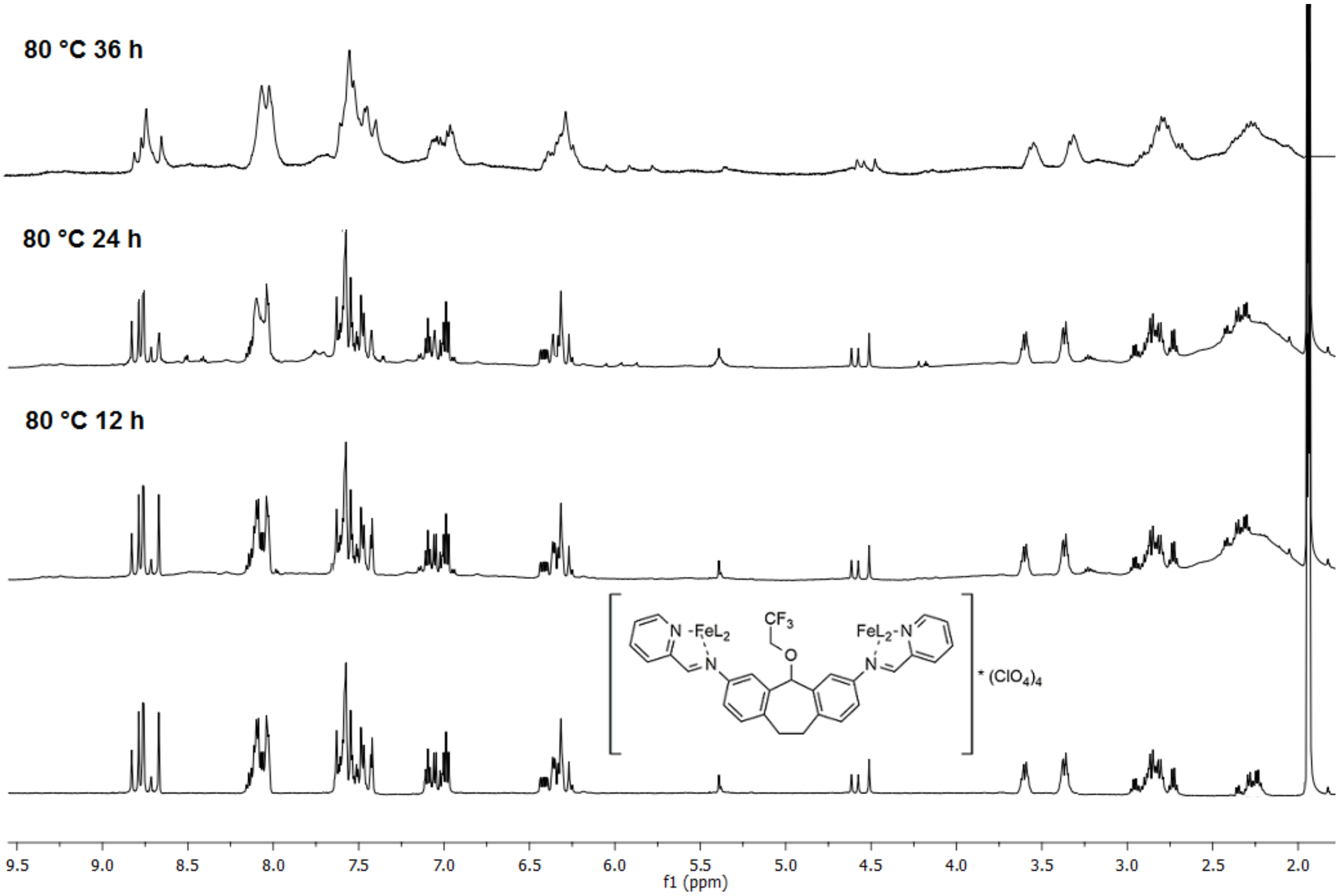

Figure S40. ^{1}H NMR spectrum of oxidation products obtained after trifluoroether cage **5** was heated at 80 °C for 36 h with 1 eq. silver perchlorate and water (CD_3CN, 600 MHz, 298 K). No substantive changes were observed in the spectrum of the cage over time at 45 °C or 80 °C.

Control Experiments

General control procedure: Control ligand **6** (7 mg, 0.02 mmol) and 400 µL of DMSO-d_6 were weighed into an NMR tube. The DMSO-d_6 was degassed with N_2 for 1h prior to attempting air free trials. Depending on the trial, 1 molar eq. of silver perchlorate (4.2 mg, 0.02 mmol), tetrabutyl ammonium perchlorate (6.98 mg, 0.02 mmol), or silver nitrate (3.4 mg, 0.02 mmol) were added to the tube. A solution of water in DMSO-d_6 (0.02 mmol, 6.4 µL of 3.17 M water in DMSO-d_6) was added to the appropriate trials. The NMR tubes were heated at 80 °C and the spectra of the reactions were taken at regular intervals. Six trials were done. The first contained 1 eq. of silver perchlorate and 1 eq. water in air, the second was done under O_2 free conditions and the third under anhydrous conditions. Trial 4 was done in the absence of silver cation (tetrabutyl ammonium perchlorate was used as a replacement), trial 5 in the absence of the

perchlorate anion (silver nitrate) and trial 6 in O_2 free DMSO-d_6 solvent only to ensure that the solvent plays no role in the oxidations. The final trial was done using 1 molar eq. of silver perchlorate, 1 eq. water and 0.66 eq. iron perchlorate in DMSO-d_6 to determine whether the presence of iron plays a part in the formation of the ketone over the alcohol.

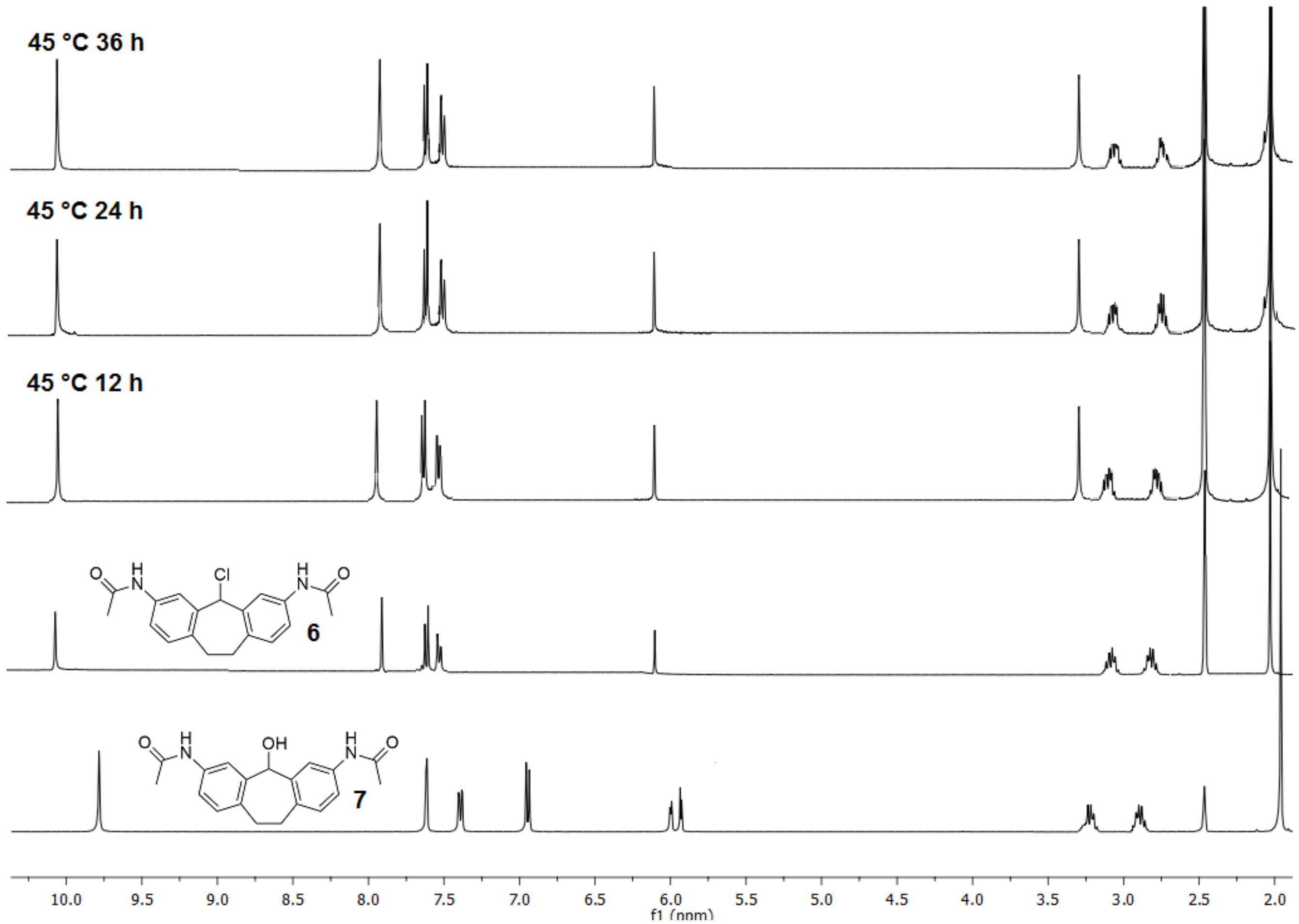

Figure S41. ^{1}H NMR spectrum of oxidation products obtained after **6** was heated at 45 °C with 1 eq. silver perchlorate and water for 12, 24 and 36 h (DMSO-d_6, 400 MHz, 298 K). No substantive changes were observed in the spectrum of the test compound at this temperature.

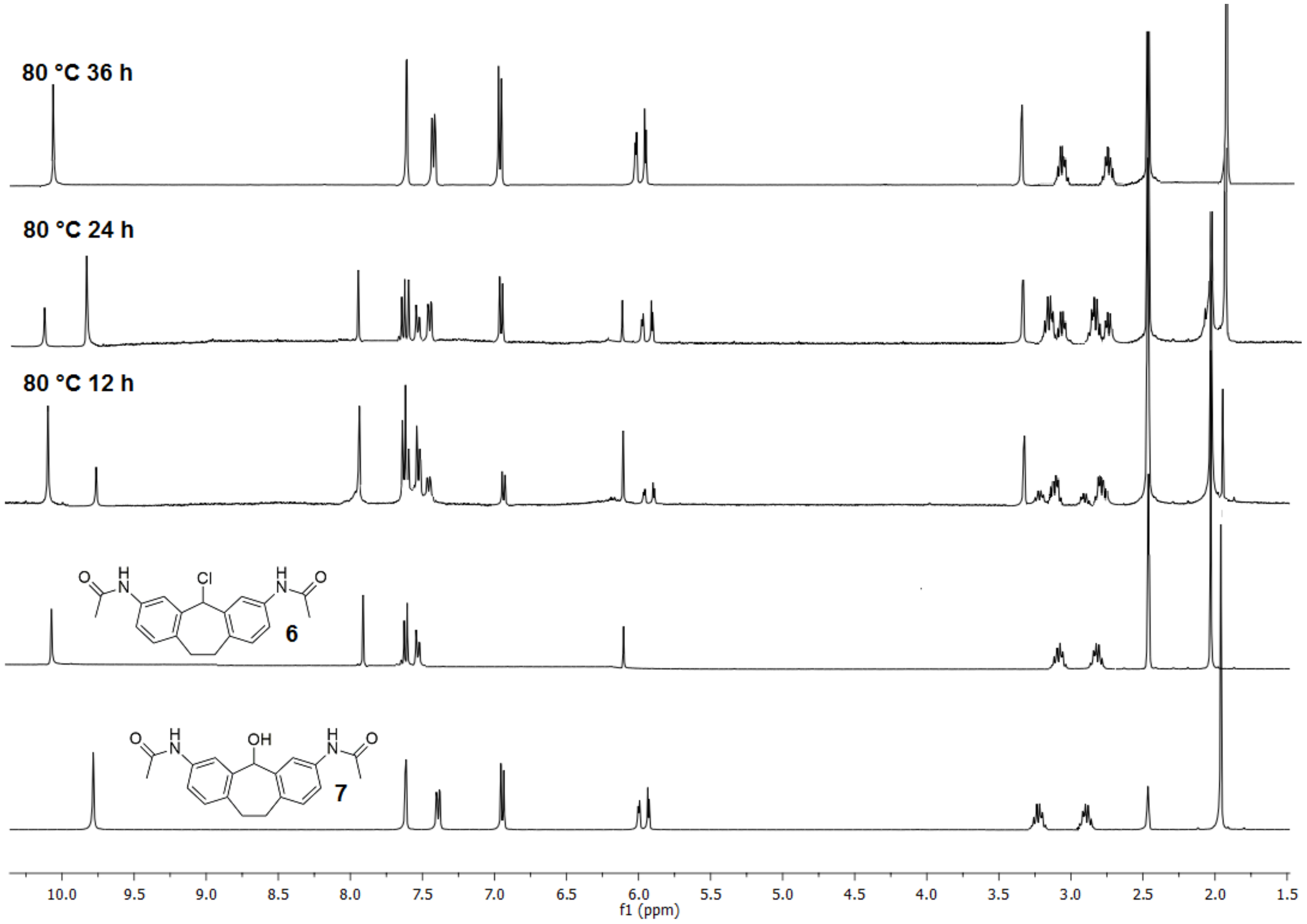

Figure S42. ^{1}H NMR spectrum of oxidation products obtained after **6** was heated at 80 °C with 1 eq. silver perchlorate and water under a nitrogen atmosphere for 12, 24 and 36 h (DMSO-d_6, 400 MHz, 298 K). The chloride test ligand was converted to the alcohol ligand within 36 h despite the absence of O_2.

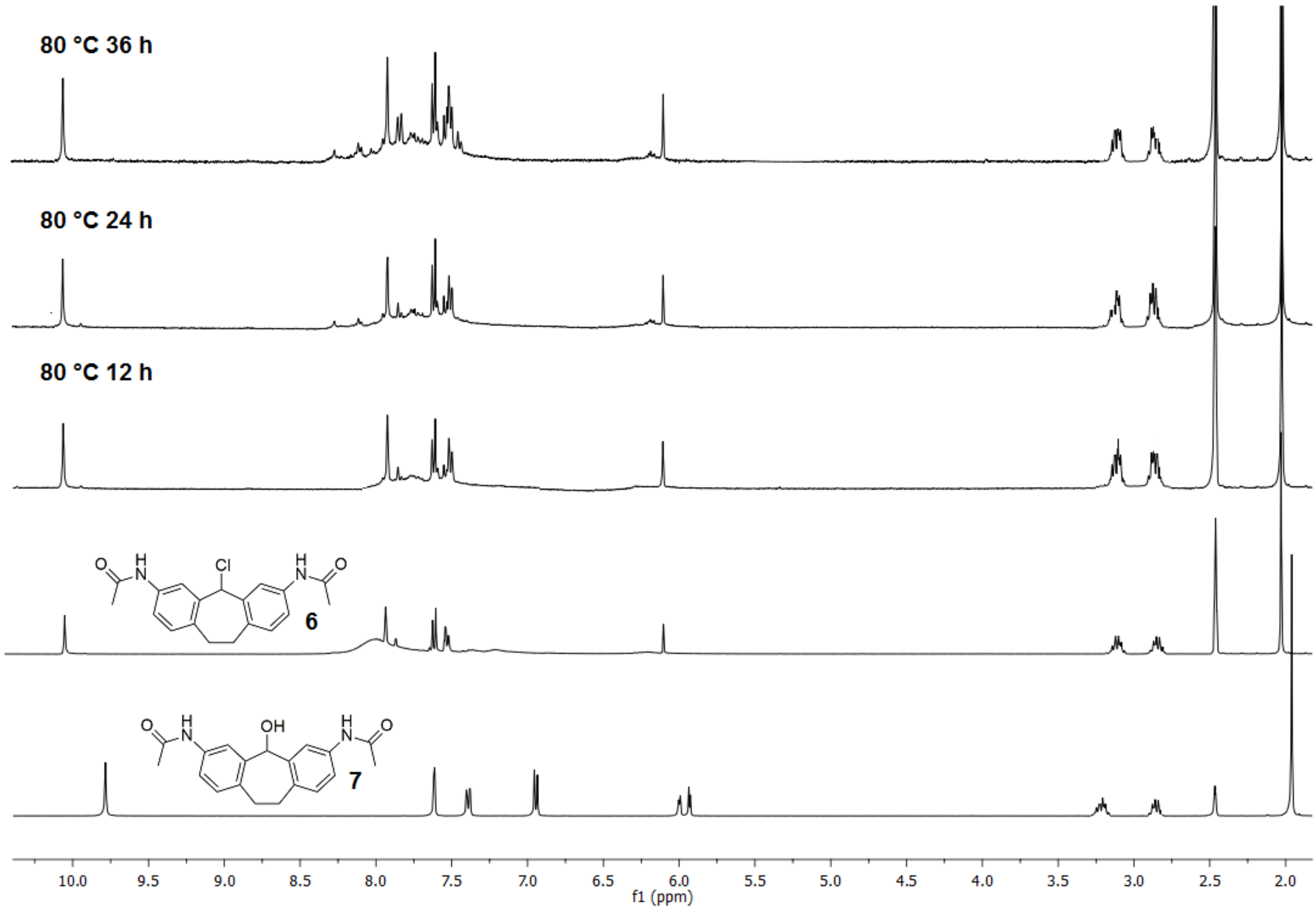

Figure S43. ^{1}H NMR spectrum of oxidation products obtained after **6** was heated at 80 °C with 1 eq. silver perchlorate under anhydrous conditions for 12, 24 and 36 h (DMSO-d_6, 400 MHz, 298 K). No substantive changes were observed in the spectrum of the test compound other than a small amount of decomposition.

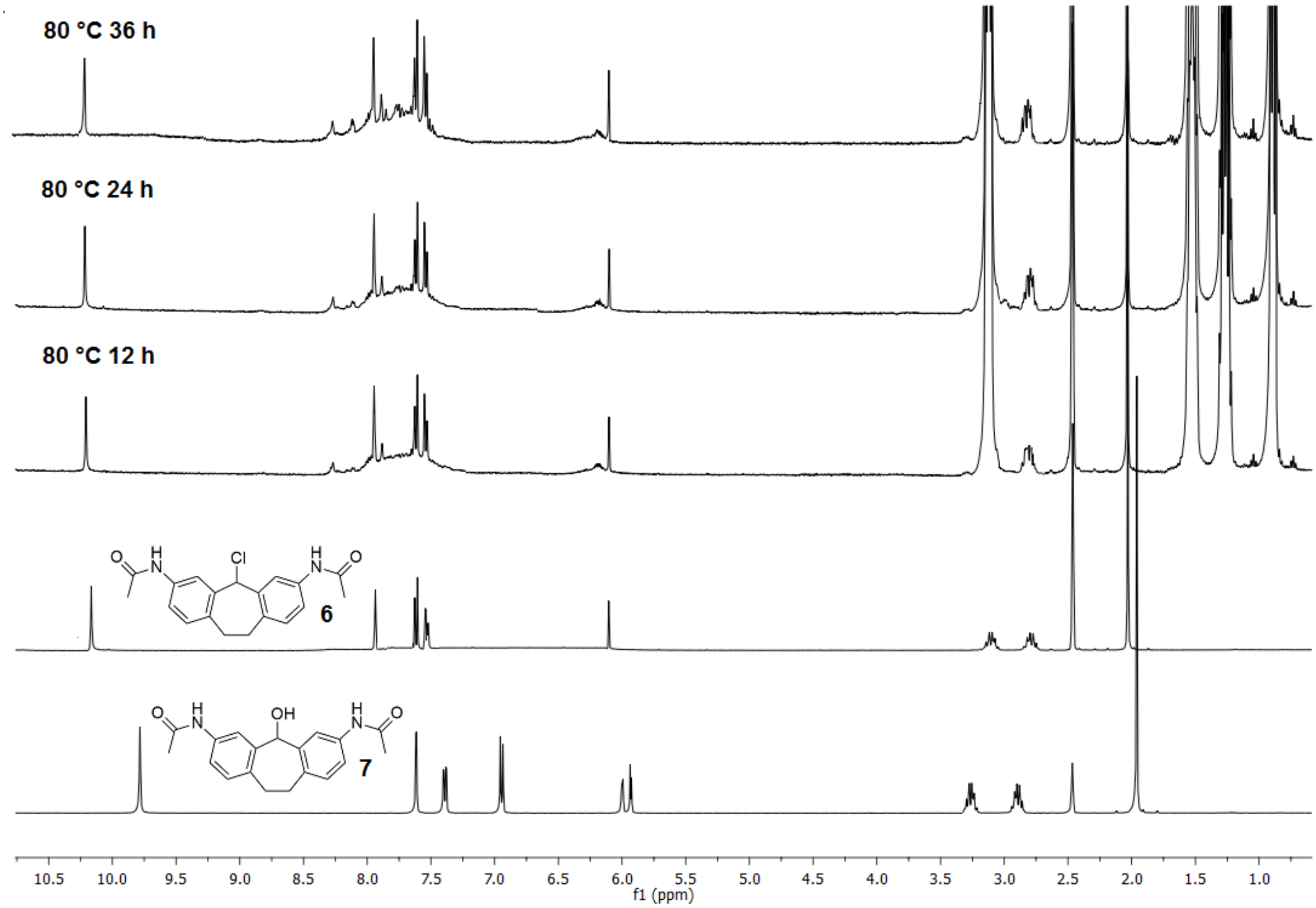

Figure S44. ^{1}H NMR spectrum of oxidation products obtained after **6** was heated at 80 °C with 1 eq. tetrabutyl ammonium perchlorate and water for 12, 24 and 36 h (DMSO-d_6, 400 MHz, 298 K). No substantive changes were observed in the spectrum of the test compound other than a small amount of decomposition.

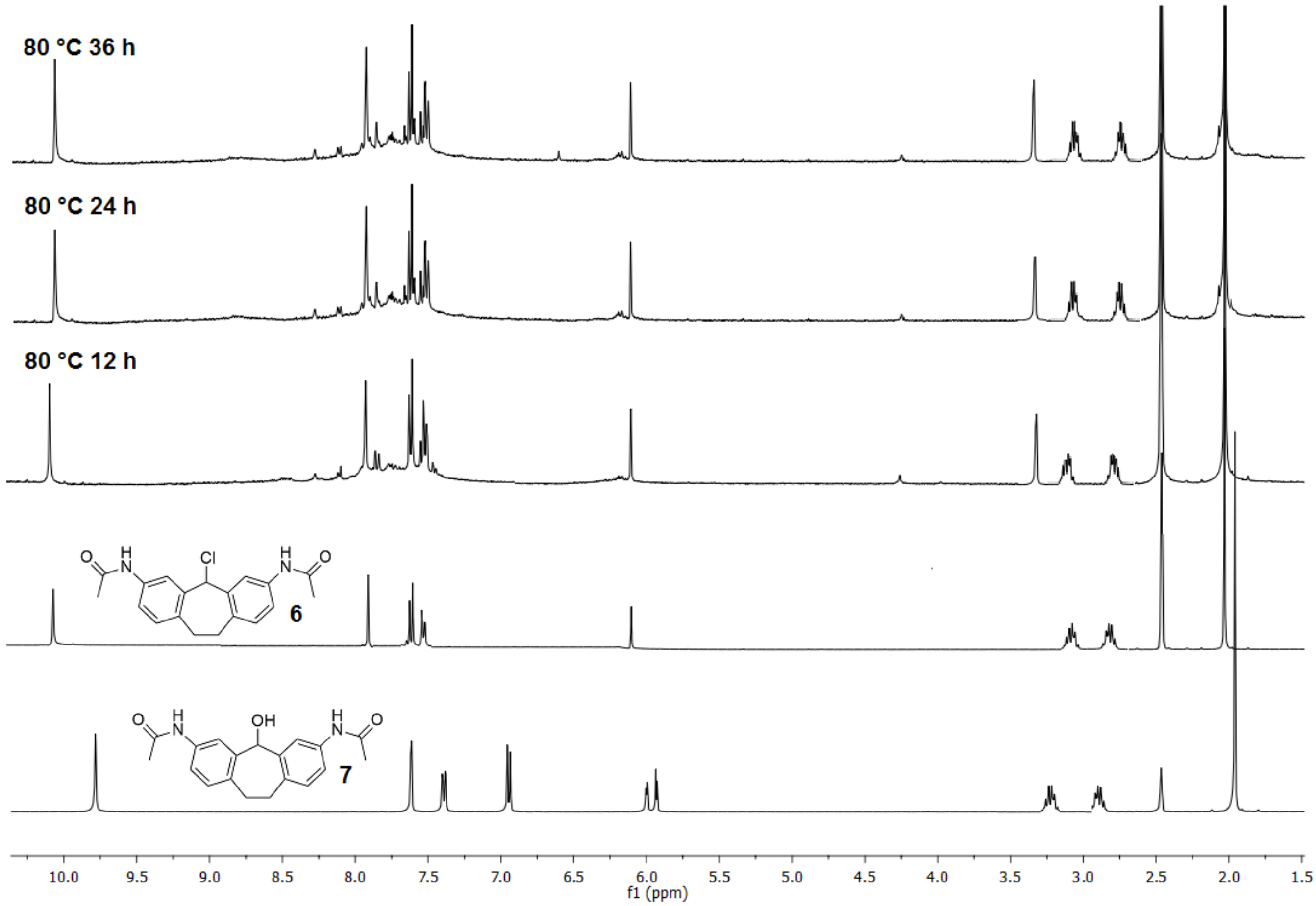

Figure S45. ^{1}H NMR spectrum of oxidation products obtained after **6** was heated at 80 °C with 1 eq. silver nitrate and water for 12, 24 and 36 h (DMSO-d_6, 400 MHz, 298 K). No substantive changes were observed in the spectrum of the test compound other than a small amount of decomposition.

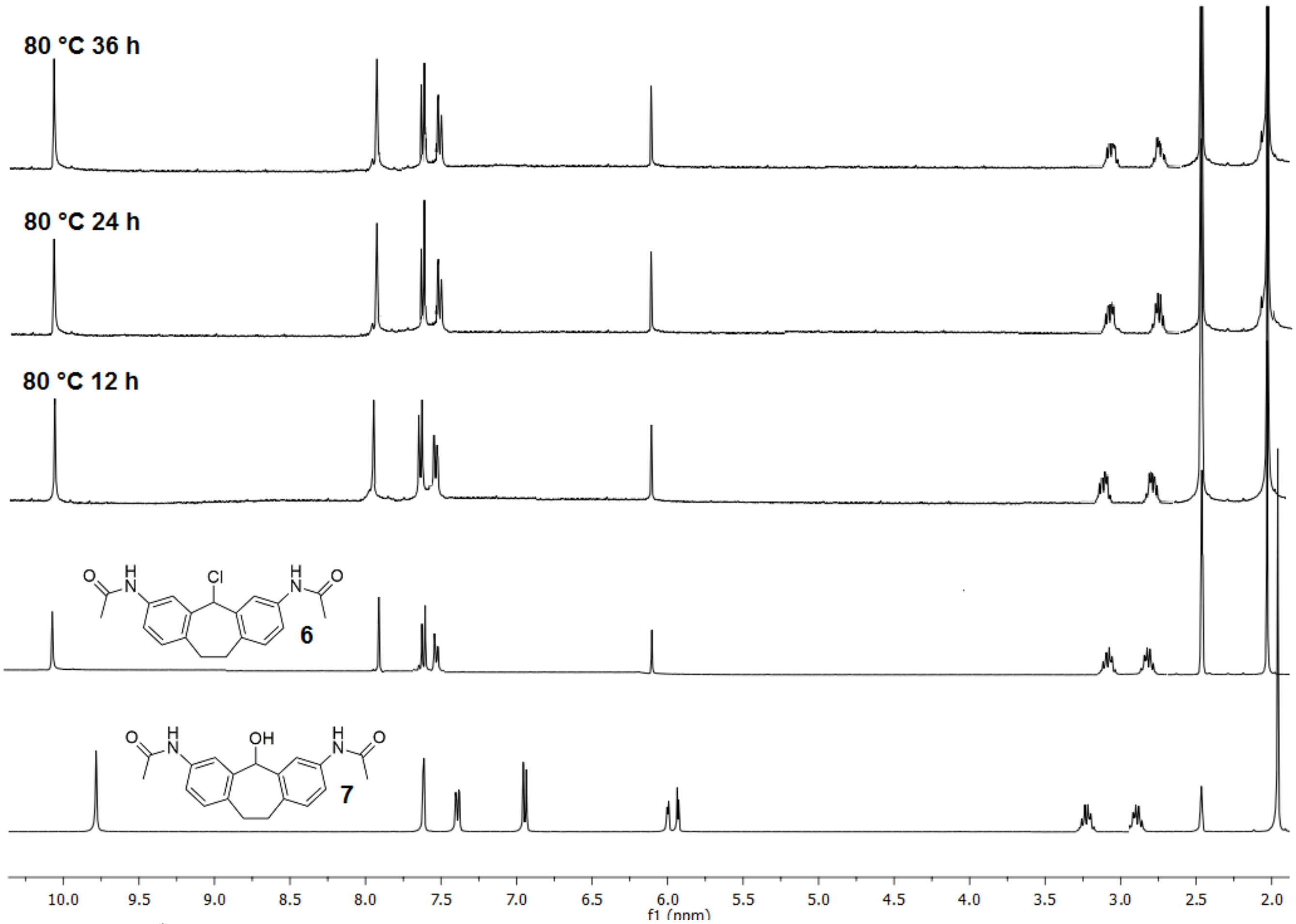

Figure S46. ^{1}H NMR spectrum of oxidation products obtained after **6** was heated at 80 °C in DMSO-d_6 solvent only for 12, 24 and 36 h (DMSO-d_6, 400 MHz, 298 K). No changes were observed in the spectrum of the compound.

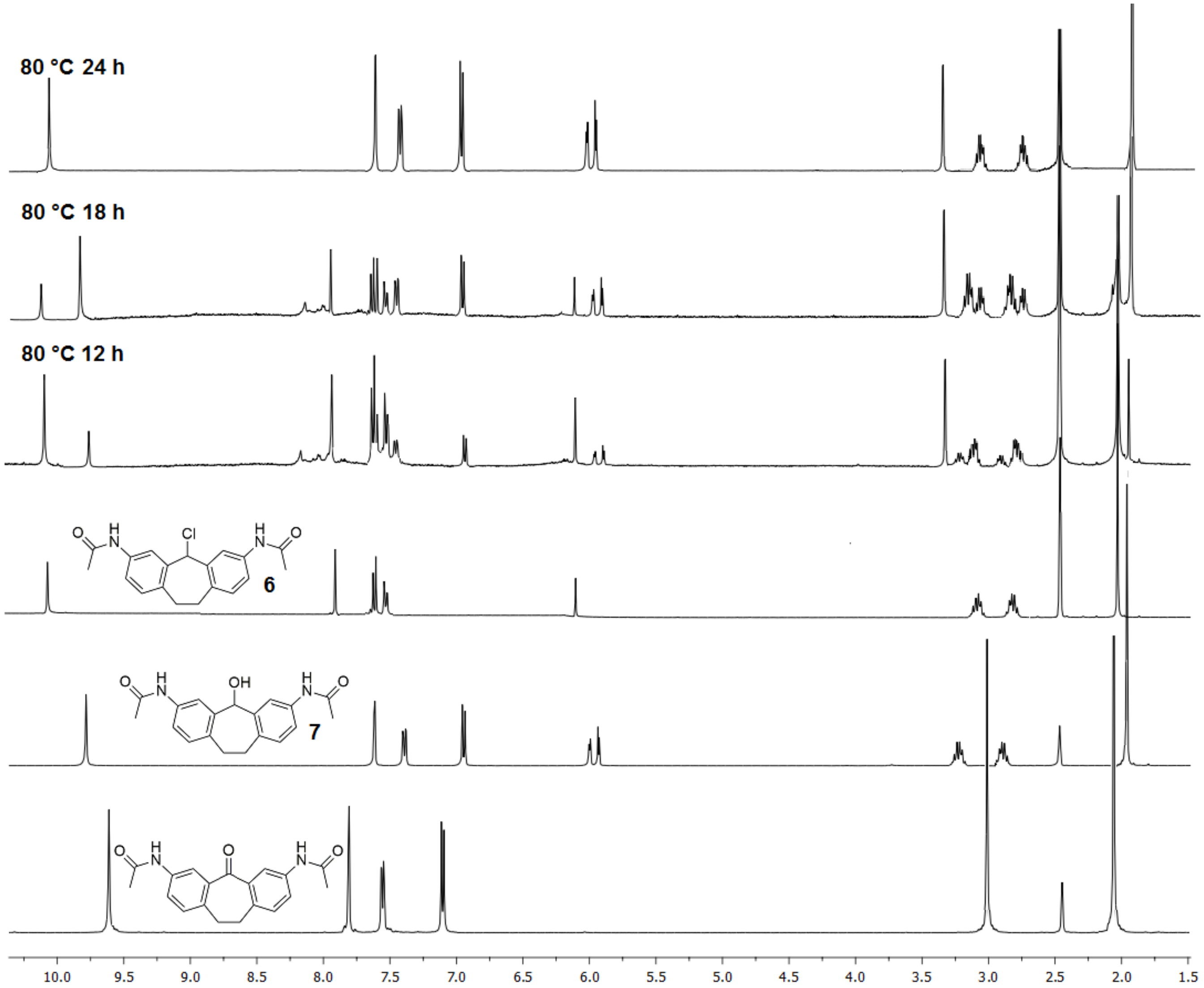

Figure S47. ^1H NMR spectrum of oxidation products obtained after **6** was heated at 80 °C with 1 eq. silver perchlorate, 1 eq. water, and 0.66 eq. iron perchlorate for 12, 24 and 36 h (DMSO-d_6, 400 MHz, 298 K). No substantive changes were observed in the spectrum of the test compound when heated at 45 °C for 24 hours. However, when heated at 80 °C, **6** was cleanly converted to **7** in 24 h with no trace of the ketone ligand present.

Structure Optimizations

Structures of the suberone complex **1** formed from ligand **A** and four possible isomers of the M_2L_3 helicate involving ligand **C** (with zero, one, two, or three chlorides pointed toward the center of the helicate) were optimized using wB97X-D/6-31G(d).[3,4] The structures of the uncomplexed ligands were optimized at the same level of theory. The relative stability of the complexes was then assessed by computing the reaction energy for the hypothetical ligand exchange:

$M_2A_3 + 3\ C <==> M_2C_3 + 3\ A$

The results indicate that all chloride complexes are less stable than suberone complex **1**. Among the chloride complexes, the isomer with all chlorides facing outward is the most stable. Each inward-facing chloride destabilizes the helicate by ~5-8 kcal/mol.

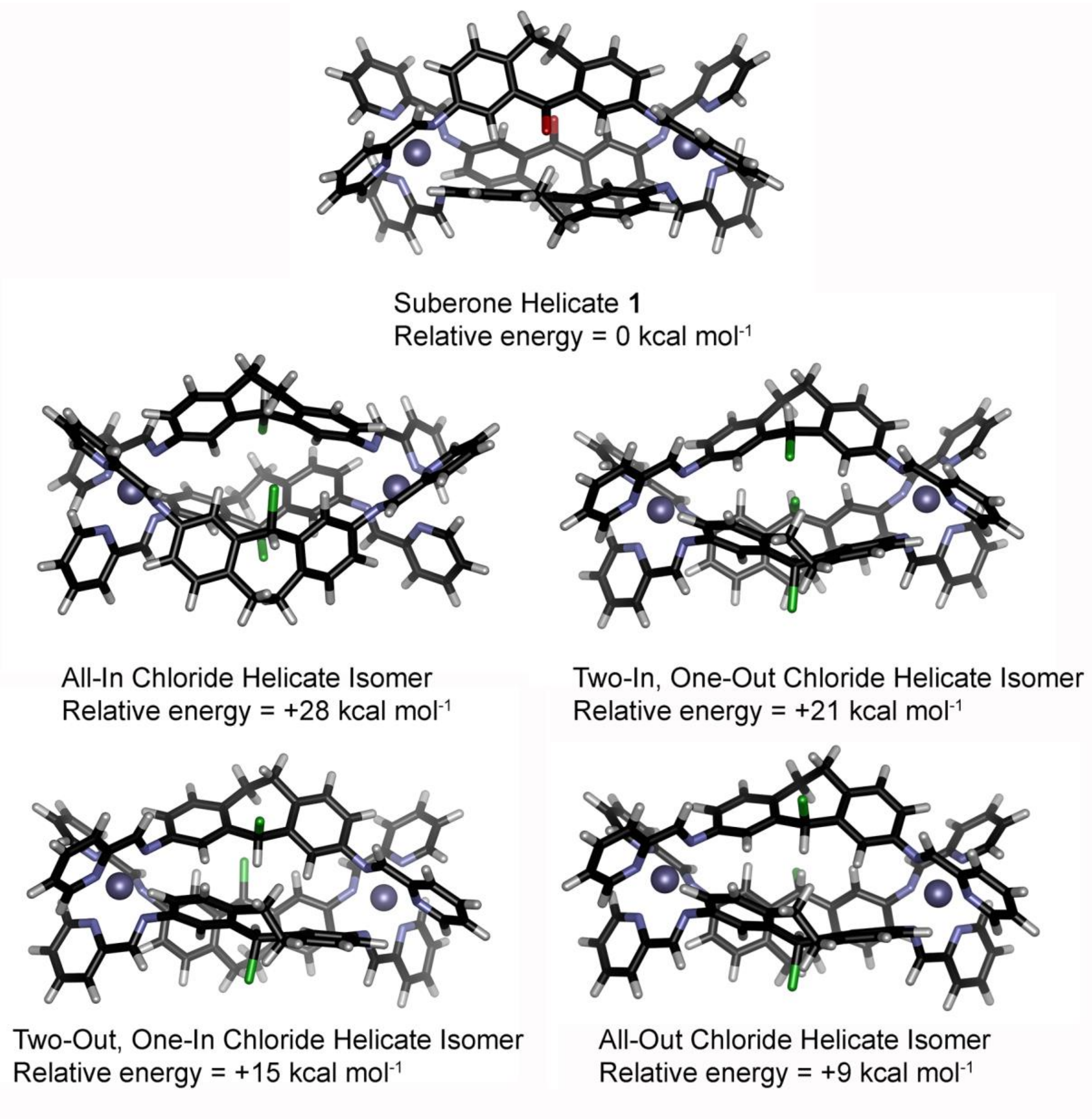

Figure S48. Optimized structures (and calculated relative energies) of suberone cage **1** and the four possible isomers of M_2L_3 cages that could be formed from assembly of **C**, $Fe(ClO_4)_2$ and 2-formylpyridine.

References

1) M. J. S. Dewar, E. G. Zoebisch, E. F. Healy, J. J. P. Stewart, *J. Am. Chem. Soc.* 1987, **107**, 3902-3909; calculations performed on SPARTAN 06, Wavefunction Inc.

2) M. C. Young, A. M. Johnson, R. J. Hooley, *Chem. Commun.* 2014, **50**, 1378-1380.

3) J.D. Chai and M. Head-Gordon, *Phys. Chem. Chem. Phys.* 2010, **10**, 6615-6520.

4) a) W. J. Hehre, R. Ditchfield, and J. A. Pople, *J. Chem. Phys.* 1972, **56**, 2257. b) V. A. Rassolov, J. A. Pople, M. A. Ratner, and T. L. Windus, *J. Chem. Phys.* 1998, **109**, 1223.
